# Charismatic Nonverbal Displays by Leaders Signal Receptivity and Formidability, and Tap Approach and Avoidance Motivational Systems

**DOI:** 10.3389/fpsyg.2020.526288

**Published:** 2020-10-22

**Authors:** Caroline F. Keating, Fiona Adjei Boateng, Hannah Loiacono, William Sherwood, Kelsie Atwater, Jaelah Hutchison

**Affiliations:** ^1^Psychological and Brain Sciences, Colgate University, Hamilton, NY, United States; ^2^Department of Psychology, New York University, New York, NY, United States

**Keywords:** charisma, leadership, nonverbal communication, politics, approach/avoidance, EEG

## Abstract

Status cues and signals act as guidance systems by regulating social approach and avoidance. Applied to leadership, we hypothesized that nonverbal displays conveying the dual-status messages of receptivity and formidability and the approach/avoidance motives they activate set conditions for charismatic, leader–follower relationships. We investigated *perceptions* of charisma, the *nonverbal signals* associated with them, the *motives* they energize, and the *relationships* they support across levels of analysis. At the social–perceptual level (studies 1a–d), eligible voters rated political leaders’ traits after viewing silent, 30-s videos of speeches presented online. As predicted, perceptions of politicians’ receptivity (warmth and attractiveness) and formidability (competence and power) were independently associated with perceptions of their charisma; perceptions of trustworthiness and authenticity showed weaker or negligible associations. Results were similar when the stimuli were female, Jamaican educational leaders. Leaders’ nonverbal behavior was linked to perceptions of their receptivity, formidability, and charisma in study 2. At the brain systems level, studies 3a and 3b tested predictions that charismatic nonverbal performances stimulate equivalent degrees of approach and avoidance motivation in observers. Brain recordings *via* electroencephalography (EEG) were made while undergraduates viewed leaders rated high or low in charisma. Discrepancies in alpha activity in the left and the right frontal hemispheres (associated with approach and avoidance, respectively) were relatively diminished when participants viewed highly charismatic political leaders, indicating that approach and avoidance motives are energized in response to charismatic performances. The EEG patterns for Jamaican leaders were similar but not significant. At the group level of analysis, study 4 sought evidence that charismatic leaders create uniquely influential relationships with followers. Video recordings of student leaders interacting with pairs of unfamiliar students during a group decision-making task were assessed for leader receptivity, formidability, and charisma by independent sets of undergraduate judges. Perceptions of student leaders’ receptivity and formidability predicted their charisma, and charismatic leaders were most influential in bringing followers to privately accept a controversial group decision. Across studies, evidence generally supported hypotheses generated from status cues theory: charismatic leadership builds upon the nonverbal projection of dual-status messages and the approach/avoidance motives they engender, setting conditions for a uniquely powerful brand of influence.

## Introduction

Like the promise and peril of a new romance, a charismatic presence is hard to resist. Charismatic individuals pique human vulnerability to their influence by exuding dual, nonverbal status messages. Their bodies transmit submissiveness and dominance, warmth and power, and receptivity and formidability, thereby activating dual approach and avoidance motivational systems in perceivers and setting conditions for uniquely potent psychological bonds. From a status cues theory perspective, the nonverbal projection of these qualities and the motives they energize comprise the body and soul of charisma (Keating, 2002, 2011, 2018)^1^. Here these ideas are applied to leadership and studied from social–perceptual, behavioral, brain system and group interaction levels of analysis.

Leaders essentially *move* people, and charismatic leaders excel in this regard (Shamir et al., 1993; Goleman et al., 2000; Riggio and Tan, 2013). Charisma, in the context of leadership, has been variously described as a gift from God (Greek translation), a disposition (e.g., Tskhay et al., 2017), a set of behaviors or values (e.g., Eagly et al., 2003; Antonakis et al., 2016), affect transferal (Erez et al., 2008; Sy et al., 2018), embodiment (Reh et al., 2017), and the signaling of traits and values (e.g., Gray and Densten, 2007; Grabo et al., 2017). Howell and Shamir (2005) emphasize charisma’s role in leader–follower relationships. They viewed charisma as “residing in the relationship between leaders who exhibit certain charismatic qualities and behaviors and those followers who have certain perceptions, emotions, and attitudes toward the leader, the group led by the leader, and the vision advocated by the leader” (Howell and Shamir, 2005, p. 98). This emphasis on interpersonal context is consistent with the construal of leadership as a relationship in which follower perceptions, identities, and social motives play crucial roles (Haslam et al., 2010). Informed by these different perspectives and guided by a status cues approach (Keating, 1985, 2002, 2011, 2018; Mazur, 1985, 2005), we investigated the *social perceptions* of charismatic leaders, the *nonverbal signals* charismatic leaders display, the *motives* activated in perceivers as they observe charismatic leaders, and the *leader–follower relationships* these processes support.

Social perceptions (studies 1a–d): The origins of charisma flow from the face-to-face nature of status relationships throughout human evolution (Van Vugt et al., 2008; Day and Antonakis, 2012; Castelnovo et al., 2017; Grabo et al., 2017). In particular, the way human nonverbal communication systems evolved enable the transfer of affect and experience between leaders and followers through mimicry, imitation, embodiment, and related identity processes (Erez et al., 2008; Wiltermuth and Heath, 2008; Anderson and Kilduff, 2009; Riggio and Riggio, 2010; Castelnovo et al., 2017; Reh et al., 2017; Knowles, 2018). Researchers often focus on the *positive* communications charismatic leaders transmit: excitement, arousal, affection, and trust (Cherulnik et al., 2001; Pastor et al., 2007; Erez et al., 2008; Sy et al., 2018). From these perspectives, charisma’s essence is in the display and stimulation of positive arousal in followers.

Charisma, however, reveals a second, equally important social complexion evident in ordinary as well as extraordinary relationships. Tskhay and colleagues studied how individuals conceptualize “everyday” charisma as a trait in themselves and others (Tskhay et al., 2017). Analyses revealed two distinct, defining, dispositional dimensions—affability and influence (Tskhay et al., 2017)—the latter including aspects of social power. In the extraordinary context of leader–follower relationships, theorists capture the “influence” dimension in their formulations of charisma by including leader dominance, competence, formidability, and anger (e.g., Castelnovo et al., 2017; Grabo et al., 2017; Reh et al., 2017). Thus, charisma in the more distal, extraordinary relationship context of leaders and followers matches the proximate, interpersonal relationship experience in the dual projection of a receptive, inviting sociable dimension and a formidable, threatening one. In leadership, as in life, the evaluative dimensions of interpersonal warmth/attractiveness and agency/power (Rosenberg et al., 1968; Judd et al., 2005) or, alternatively, warmth and competence (Fiske et al., 2007) are fundamental to social perceptual space.

In the studies presented here, we combine warmth and attractiveness as component trait measures of *receptivity* and competence and power as components of *formidability*. We relied on these traits because (1) they appear frequently in the impression formation literature and (2) they conjure both psychological (i.e., warmth, competence) and physical (i.e., attractiveness, powerfulness) aspects of social perceptions. Including both aspects seemed truer to the evolutionary roots of our arguments and is supported by research that confirms the importance of physical formidability to status-related judgments (e.g., Lukaszewski et al., 2016). In addition, we pitted these perceptions against compelling alternatives, trustworthiness and authenticity, as a check on whether perceptions of charisma are simply generally positive. *From a status cues perspective, Hypothesis I was that perceivers’ impressions of leader receptivity and formidability would independently contribute to perceptions of leader charisma (studies 1a–d).*

Nonverbal behavior (study 2): How do nonverbal behaviors carry dual receptivity/formidability messages? Cross-species research suggests that the art of the signaling deal relies on cues and behaviors that are graded in intensity and meaning. Unambiguous messages, such as full-blown aggressive displays or unmitigated signs of appeasement, are potentially costly in that they leave both signaler and recipient few options to change course (Dawkins and Krebs, 1978). Signaling strategies are instead often nuanced and multifaceted, thereby offering opportunities for gradual escalation or de-escalation. Similar to other species, for example, human courtship behavior both invites and discourages approach (Moore, 2010), and the physiognomic cues of preferred lovers and mates simultaneously convey mixed-status messages (Keating and Doyle, 2002). Political candidate allure may be based on similar “come hither but beware” nonverbal contrivances.

Leadership theorists essentially describe dual-status signals but from a different perspective. Some contend that successful leaders engender *psychological closeness* and *distance* in followers (e.g., Shamir, 1995, 2013; Kark et al., 2003; Razin and Kark, 2012; Antonakis and Jacquart, 2013). The benefits of signaling closeness are easy to explain; it fuels liking, motivates proximity, stimulates emulation of the leader, and enhances the fusing of leader–follower identity and group identity (Hogg, 2001, 2014; Kark et al., 2003). What might be the benefits of signaling psychological distance? Psychological distance helps preserve the social hierarchy that defines high status as distinct and deserving of deferential treatment and special access to resources (Liberman and Trope, 2008; Shamir, 2013). The specialized titles and rules of interaction to indicate rank among military personnel are examples (Shamir, 2013). Distance can also be used to cloak weakness and failure by keeping idealized images most salient, for example, by having surrogates perform mundane tasks while leaders perform the extraordinary kind (Shamir, 2013). Signaling psychological distance makes leaders seem formidable by proffering unique, celebrity status. Thus, leaders strive for the right balance of signaling psychological closeness to and distance from followers (Shamir, 1995, 2013; Razin and Kark, 2012; Bligh and Riggio, 2013).

Political leaders’ nonverbal displays are therefore likely to comprise mixed receptivity/formidability signals (e.g., open arms with palms up and brows raised followed by lowered brows and pointing) or dual messages simultaneously embedded within the same action (e.g., one hand in a pocket while pointing with the other hand). However, in natural, ecologically valid leadership settings, the multitude of ways in which nonverbal behaviors are combined and performed makes predicting their elements and specific meanings difficult. Hertenstein (2011) describes two principles of tactile communication that are relevant here. *Equifinality* describes the idea that different nonverbal behaviors can serve the same communicative function (Hertenstein, 2011, p. 301). For example, the display of fists and lowered brows both signal aggression. *Equipotentiality* is the idea that the same gesture can assume different meanings; placing an arm over another’s shoulder can convey intimacy or dominance (Hertenstein, 2011, p. 301). Gaze projects either intimacy or threat, depending on the context (Adams and Nelson, 2016), and expansive postures have been linked to perceptions of both attractiveness and dominance (Vacharkulksemsuk et al., 2016). Thus, we anticipated that many nonverbal behaviors would be associated with *both* receptivity and formidability perceptions and with charisma.

For these reasons, we deployed a basic “bottom up” approach (Buck and Miller, 2016) to track nonverbal messages in study 2. We content-analyzed short, silent video recordings (thin slices) of political speeches to identify common gestural and gaze behaviors, recorded their frequencies, and computed correlations between behaviors and perceptions of leaders’ receptivity, formidability, and charisma. Parallel to predictions for social perceptions, *Hypothesis II was that nonverbal behaviors associated with both receptivity and formidability would also be associated with impressions of charisma.*

Approach/avoidance motivational systems (studies 3a and b): Signaling systems and the perceptions they generate do the work of regulating approach and avoidance, the very foundation of social life (Strack and Deutsch, 2004; Buck and Renfro Power, 2006; Krieglmeyer et al., 2010). This makes sense in evolutionary context: social perceptions enhance individual fitness by helping organisms quickly assess important capacities and qualities of potential social targets, thereby guiding approach and avoidance (Schaller, 2008). Moreover, conceptual, evaluative space has parallels in motivational space, suggesting that concepts and motives likely evolved in complementary fashion (Delton and Sell, 2014). Depending on the theorist, perceptions of attractiveness, submissiveness, warmth, or receptivity motivate approach and perceptions of power, dominance, competence, or formidability motivate avoidance (e.g., Rosenberg et al., 1968; Keating, 1985; Judd et al., 2005; Fiske et al., 2007; Schaller, 2008). If charisma embodies these dual messages, then its expression should energize two motivational systems in perceivers: approach and avoidance. Unique to our status cues model, dual perceptions *and* motives are projected to fuel relationships between charismatic leaders and followers (Keating, 2011, 2018).

We sought implicit and explicit measures of perceivers’ internal, motivational orientations toward charismatic leaders. Our implicit measurement was captured from brain activity recordings taken as perceivers viewed thin slices of leader displays. Brain hemispheres are differently associated with approach and avoidance motivation. At the brain systems level of analysis, activation of the left frontal cortical region is associated with approach, while activation in the right frontal cortical region is linked to avoidance (Sutton and Davidson, 1997; Amodio et al., 2004; Harmon-Jones et al., 2010; Kelley et al., 2017).

In their review, Harmon-Jones and Gable (2017) present extensive evidence that approach/avoidance motivational systems reflect more positive vs. negative affective valence. Specifically, anger is typically considered to be negatively valenced but registers in the approach-related, left hemisphere, thereby potentiating approach action (aggression). Thus, tracing the relative activation of each hemisphere indicates likely motivational direction toward or away (Harmon-Jones and Gable, 2017), as receptivity and formidability signals engage each hemisphere, not one or the other.

Electroencephalography (EEG) is commonly used to measure motivation-related brain activity across hemispheres (Harmon-Jones and Gable, 2017). Boksem et al. (2012) used EEG to investigate whether participants primed to feel powerful registered more left-hemisphere (approach) brain activity than participants primed to feel powerless. As predicted, alpha activity (8–13 Hz), which is associated with the suppression of response, was lowest in the left frontal hemisphere of the participants primed to feel powerful, presumably unleashing approach (Boksem et al., 2012). These researchers concluded that instilling feelings of power or powerlessness in participants produced differential patterns of brain activation linked to approach/avoidance motivations (Boksem et al., 2012).

We anticipated differential activation patterns in perceivers presented with charismatic vs. non-charismatic leaders. If charismatic displays elicit both approach and avoidance motivations in perceivers, then EEG recordings of alpha power, a measure inversely related to brain activity, should be equivalent in each hemisphere as participants view charismatic leaders but asymmetric when they view leaders low in charisma. In essence, non-charismatic leaders are expected to generate too much *relative* signal (i.e., alpha power) from either the approach or the avoidance hemisphere, undermining a charismatic balance of approach (come hither) and avoidance (beware) motivations. We found no EEG alpha power research on this possibility, but using a different brain recording technique (functional magnetic resonance imaging), Spezio and colleagues discovered greater activation of specific brain regions in perceivers’ reactions to viewing the faces of losing than winning political candidates (Spezio et al., 2008). Winning candidates generated negligible activation in perceivers. The researchers speculated that negative appraisals likely drove the effect by outweighing positive ones. We, too, sought evidence of such imbalance, but within the brain’s motivational systems.

We propose that non-charismatic performances tip the motivational scale in one direction or the other, whereas charismatic performances balance approach/avoidance motivations. Two EEG studies (3a and 3b) were conducted to test this idea on the nonverbal displays of political leaders and Jamaican educational leaders. Participants wearing electrode nets watched subsets of thin slices showing leaders who scored low or high in charisma (as determined in study 1). Our implicit motivational measure was spectral power in the alpha-band frequency range (8–13 Hz) in the left (approach) and the right (avoidance) frontal cortex. Alpha is typically understood as inhibitory and therefore inversely related to regional, frontal cortical activity. What is inhibited depends upon the hemisphere; specifically, high levels of alpha in the left hemisphere suppress approach, whereas high levels in the right hemisphere dampen avoidance (Klimesch et al., 2006; Boksem et al., 2012; Harmon-Jones and Gable, 2017). Difference scores between alpha activity in the left and the right hemispheres recorded by specific electrodes are used to tap asymmetric activity that presumably reflects the dominance of one motivational tendency over the other (Harmon-Jones and Gable, 2017). Based on this understanding, alpha should show its greatest imbalance in response to non-charismatic compared to charismatic performances, regardless of absolute levels of alpha.

We added explicit measures of leader charisma to both EEG studies. In each, the participants indicated the likelihood that they would consider voting for a leader after each thin slice presentation.

Hypothesis III was that non-charismatic performances would activate the left and the right frontal hemispheres unevenly (i.e., more of one than the other) and correspond with relatively low voter support, while charismatic performances would activate them evenly and correspond with enhanced voter support.

Leader–follower relationships: At the level of the group, why would a combination of approach/avoidance motivations stimulated by charismatic leaders render followers especially susceptible to their influence? At the extremes, leaders who deploy distance-generating rituals, such as harsh hazing in combination with initially welcoming treatment, are able to transform new group members into highly devoted followers (Galanter, 1999; Keating et al., 2005). Similarly, successful indoctrination techniques typically punctuate harsh or brutal treatment with signs of acceptance and warmth (Baron, 2000). Nothing may be quite as potent as the activation of mixed approach/avoidance motives when it comes to stimulating attachment generally, as cross-species, developmental science shows (e.g., Harlow, 1959; Rajecki et al., 1978; Cairns, 1979; Bowlby, 1982). To varying degrees, charismatic leaders may be beneficiaries of the tendency social organisms have to bond with, follow, and become dependent upon on agents who alternately accept (reward) and reject (punish) them. The truism seems to be that humans come to love what they sometimes suffer for.

Does this pattern hold for charismatic leadership? A study of charismatic or transformational leadership consistent with the known effects of stimulating these signaling and motivational dualities was performed by Kark and colleagues (Kark et al., 2003). These researchers studied 888 bank employees who assessed their identification with branch managers and work units and then rated their managers/unit leaders for transformational leadership qualities that included aspects of charisma. The main result showed that employees who had transformational (charismatic) leaders were both strongly empowered through close identification with the leader and highly dependent on the leader’s personal power and approval (Kark et al., 2003). Charisma, it seems, manifests an aura of both delight and danger by engaging a psychological pull (closeness/attraction) and push (distance/avoidance), thereby potentiating intense levels of attachment and devotion. That balance taps approach and avoidance motivational systems and, in doing so, is experienced as “charismatic” by followers^2^. In an on-campus, laboratory study (study 4) of leader–follower interactions, *Hypothesis IV was that campus leaders perceived as charismatic (receptive and formidable) would be most influential in bringing followers to publicly and privately accept a controversial group decision.*

All studies in this series were reviewed and approved by the university Institutional Review Board.

## Studies 1A Through 1D: Social Perceptions

Overview: Four, online studies confirmed Hypothesis I, that leaders perceived as charismatic project receptivity and formidability. These studies also provided a basis for identifying political leaders perceived as unusually high or low in charisma for use as stimuli in studies 3a and b.

For each study, samples of US citizens of voting age were recruited *via* Amazon Mechanical Turk (MTurk). MTurk samples have both their shortcomings and strengths (e.g., Buhrmester et al., 2011; Crump et al., 2013) but are generally considered more representative of voting populations than samples of college undergraduates. We followed best practices, inserting bot checks at the beginning of each survey and deploying Turk Gate to avoid duplicating respondents. A debrief, thank you message and lab contact information concluded each study session. The participants received Amazon coupons worth $2.50–$3.00 as an incentive, depending upon the number of stimuli per study.

In each study, 30-s silent video clips or “thin slices” of leaders giving speeches were shown to the participants who rated charisma, warmth and attractiveness (receptivity), competence and power (formidability), and trustworthiness or authenticity as rival, alternative explanations. Ratings for a politician were excluded from data analyses if the participant affirmed that they recognized the politician and correctly (or nearly correctly) responded by typing the name of the politician in an open-ended query box (see Supplementary Material for details).

Factors that possibly moderate these hypothesized relationships include leader gender, party (Democrat or Republican), and speech topic (domestic or foreign affairs). To focus the reporting of our multi-study results on the main hypotheses, the ancillary analyses of these potential moderators are reported in Supplementary Material.

## Study 1A: Perceptions of US Political Leaders

### Materials and Methods

#### Participants

Data were gathered using an online Qualtrics survey distributed to US citizens of voting age (*N* = 364) through Amazon Mechanical Turk (MTurk) in early 2015, prior to the political high season of the US presidential primary. Portions of our sample recognized two political leaders (Marco Rubio and Michelle Bachmann), and there was scattered recognition of other political leaders. When data were analyzed only from respondents who did not recognize particular politicians, *n*s varied from 222 and 352, depending on the political leader.

Eighty-one percent of respondents claimed to have been registered to vote. The sample was 55.5% male and 78% White, 5% African American, 7% Hispanic, 9% Asian American, with 2% other or not reported. The modal age bracket (accounting for 41% of the sample) was between 26 and 34 years, with 28% between 35 and 54 years, 8% 55 years and older, and 23% between 18 and 25 years. Relative to actual voting populations, young White males were over-represented.

#### Stimuli

The stimuli were derived from digitally recorded speeches (available online) by male and female Democrats (D) and Republicans (R) elected to the US Senate or Congress. The main selection criteria were these: each leader had to have produced two recent speeches, one focused on domestic affairs and the other focused on foreign affairs; speakers had to be in business attire, shown from the waist up, standing behind a podium; and the quality of the videos had to be good. iMovie was used to create thin slices of 30-s lengths. The audio track was removed from each, and C-SPAN banners and logos were cropped out where needed. Qualtrics was used to share the stimuli with participants through Amazon MTurk. Editing ensured that each clip omitted pleasantries and began just after the first mention of either a foreign or domestic issue (following ten Brinke et al., 2015). Without sound, the edited clips highlighted visual cues and cloaked the topic of discussion (see Supplementary Material for additional details).

#### Procedure

The study was introduced as “snap judgments” of political leaders. Each participant viewed eight thin slices (of either foreign or domestic affairs speeches) in random order. After viewing each one, they used sliding scales to rate their impressions of charisma, warmth, attractiveness, power, competence, and trustworthiness (presented in random orders). The scales were minimally marked (1 = “not at all” to 7 = “very”), and each appeared on a separate “page” that disappeared after a response was recorded to reduce carry-over effects. After responding to trait scales, the participants reported whether they recognized the person rated and, if so, recorded that leader’s name. They also identified (or guessed) the party affiliation of each leader. The participants recorded their political identity and issue concerns. The survey took between 9 and 24 min to complete.

### Results and Discussion

Participant was the unit of analysis. Based on *a priori* thinking, the participants’ warmth and attractiveness ratings were averaged to form receptivity scores, and competence and power ratings were averaged to create formidability scores for each leader. Cronbach’s alpha for each composite score constructed for each leader ranged from 0.56 to 0.75. These scores plus trustworthiness ratings formed the bases of predictor sets in regression models, with charisma ratings as the outcome variable.

Because each participant made multiple ratings of the key variables for multiple politicians, there was non-independence due to both participant and target (politician). Additionally, due to the exclusion criteria applied, there were different sample sizes for each politician rated. Linear mixed-effects models account for non-independence in the data and permit the assignment of greater weight to estimates made from a larger number of observations. For these reasons, we used mixed-effects models to examine relationships among receptivity, formidability, trustworthiness, and charisma to test for the potential moderating effects of political leaders’ gender, party, and speech content (see Supplementary Material for a description of the rationale).

First, charisma was simultaneously regressed on formidability, receptivity, trustworthiness, and their interactions. The influence of each predictor variable could vary based on participant and/or target (political leader), so we initially included random-effects estimates that allowed the intercept and each slope to vary (separately) by participant and by politician. Estimating this model resulted in a convergence error that suggested the model was too complex for the data. However, removing the random effects of trustworthiness and formidability due to politician (their estimates were essentially zero) allowed the model to successfully converge. The fixed effects are interpretable as partial slopes from a regression. The inclusion of the random effects allowed an assessment of whether the results generalized across politician and participant.

The results from this model are presented in Table 1 and generally support Hypothesis I. Across participant and politician, the results show that both receptivity and formidability uniquely contribute to charisma (*ps* < 0.001). The unique relationship between trustworthiness and charisma was also significant (*p* < 0.05). There were no significant interactions. Three separate additional models included politicians’ gender, party, and speech content as potential moderators of these relationships; none of them moderated relationships from the first model (see Supplementary Material for study 1a for details).

**TABLE 1:** Linear mixed-effects model predicting charisma for US political leaders (study 1a).

Fixed effects	*B*	*df*	*T*	Random effects	Variance
Intercept	3.9	10.7	82.15	Intercept: target	0.36
Receptivity	0.44	17	11.34***	Receptivity: target	0.08
Formidability	0.54	355.1	18.93***	Intercept: participant	0.51
Trustworthiness	0.05	338.3	2.04*	Receptivity: participant	0.24
Receptivity × formidability	0.01	801.6	0.62	Formidability: participant	0.29
Formidability × trust	–0.01	969.8	–0.77	Trustworthiness: participant	0.27
Receptivity × trust	0.01	1,474	0.42		
Receptivity × formidability × trust	–0.01	245.8	–1.34		

*p < 0.05, **p < 0.01, ***p < 0.001.

## Study 1B: Perceptions of US Senators

Study 1b was a replication of study 1a using a larger sample of political leaders and participants.

### Materials and Methods

#### Participants

A sample of 618 US citizens of voting age participated in an online (MTurk) study in the fall of 2016 during the 3 weeks prior to the US Presidential election (Clinton vs. Trump). The modal age category was 26–34 (42% of the sample); ages ranged between 18 to over 65 years. The sample identified as 53.3% male, 71.9% White, 9.6% African American, 5.7% Hispanic, 6.6% Asian American, and the rest “other” or unidentified. Participants identified more with the Democratic (*M* = 3.90, *SD* = 2.14) than the Republican Party (*M* = 2.56, *SD* = 1.89) (on 5-point scales). In comparison to typical voting populations, the sample was skewed male and young. Rules of exclusion for ratings reduced *N* to between 618 and 566.

#### Stimuli

Video stimuli were captured from statements made from the floor of the US Senate to standardize them (adapted from ten Brinke et al., 2015). The original videos were extracted from the personal YouTube channels of 24 senators randomly chosen (with restriction; see Supplementary Material for study 1b) from the 113th and/or 114th Senate. As in study 1a, senators were included if we found two different speeches, one on foreign affairs (e.g., North Korean sanctions) and another on domestic issues (e.g., healthcare).

**FIGURE 1:**
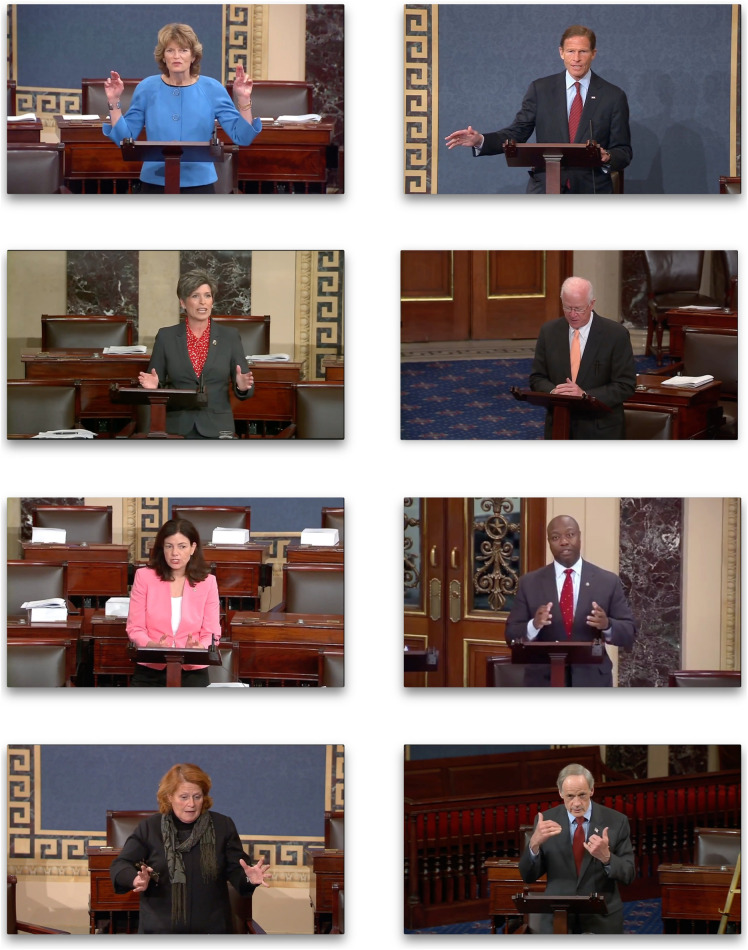
Still frames from a subset of political leaders’ thin slices used in study 1b.

The videos were downloaded and edited using iMovie to create 30-s thin slices as was done in study 1a. To prevent boredom and fatigue, the stimuli were divided into subsets, and each participant rated one subset of 12 different leaders. Rating scales were identical to those used for study 1a to render perceptions. Still frames from a sample of thin slices appear in Figure 1.

#### Procedure

The study was introduced as “first impressions of leaders” and took between 12 and 50 min to complete (*M* = 27 min; *SD* = 11.31 min). After responding to the online consent form, the participants read a 100-word opinion piece and answered four true/false questions to check that it had been read^3^. Next, they viewed and rated leaders on the randomly presented attributes. After rating each leader, the participants were asked whether they recognized them and, if so, to record their name. Lastly, the participants addressed a series of questions asking them to identify issue concerns and political affiliation and to report basic demographic information (age, gender, race, and voter registration).

### Results and Discussion

Participant was the unit of analysis, and the rules for exclusion of ratings (based on recognition criteria) were the same as for study 1a. As before, *a priori* decisions guided the construction of two composite scores, receptivity (mean of warmth and attractiveness; α = 0.90) and formidability (mean of power and competence; α = 0.83). Trustworthiness was again analyzed as a competing variable. The variable to be explained was charisma.

We used the same approach to model building as was used in study 1a, including all main effects and interactions. Estimating this model resulted in a convergence error, indicating that the model was too complex for the data. Thus, the near-zero random effect of receptivity due to target (politician) was removed to allow the model to successfully converge. With all main effects and all remaining interactions included in the model, receptivity and formidability contributed unique, positive, and significant explanatory power to charisma; this time, trustworthiness did not (see Table 2). These relationships held across politicians and participants. As for study 1a, additional models probed the potential moderating effects of political leaders’ gender, party, and speech content. There were no effects for gender and few other effects. Results for these ancillary models are reported in Supplementary Material for study 1b.

**TABLE 2:** Linear mixed-effects model predicting charisma for US senators (study 1b).

Fixed effects	*B*	*df*	*T*	Random effects	Variance
Intercept	0.23	490.5	0.99	Intercept: politician	0.15
Receptivity	0.55	1,712	5.93***	Formidability: politician	0.05
Formidability	0.21	921.6	4.74***	Trustworthiness: politician	0.02
Trustworthiness	–0.02	1,289	–0.30	Intercept: participant	0.45
Receptivity × formidability	0.01	1,851	0.38	Receptivity: participant	0.24
Formidability × trust	0.03	1,465	1.87	Formidability: participant	0.25
Receptivity × trust	–0.01	2,024	–0.56	Trustworthiness: participant	0.19
Receptivity × formidability × trust	0.00	1,520	–0.38		

*p < 0.05, **p < 0.01, ***p < 0.001.

Overall, the main results of studies 1a and 1b were consistent and supported Hypothesis 1: impressions of receptivity and formidability independently contributed to perceptions of charisma.

## Study 1C: Perceptions of Potential US Political Candidates for the 2020 Elections

Study 1c expanded on the two previous studies in several important ways: (1) stimuli were derived from speeches given by political leaders identified in the news media during the spring of 2019 as potential candidates or running mates for the 2020 US presidential election, (2) we assumed leaders to be recognizable and included all completed protocols in the analyses, omitting questions about recognition, (3) “authenticity,” rather than trustworthiness, was included as a rival explanatory variable, (4) the participants rated leaders on receptivity and formidability directly (not on their component traits), (5) the participants rated just one of four traits (receptivity, formidability, authenticity, or charisma), and (6) the unit of analysis was political leader.

### Materials and Methods

#### Participants

A total of 294 MTurk participants who were US citizens and of eligible voting age submitted responses: 178 males, 110 females, and six other/unspecified. Reported racial identification was 207 White, 36 Black, 16 Asian, one Native American/Pacific Islander, 13 Latinx, and 21 unspecified. Ages ranged from 19 to 72 years (*M* = 42.15, median = 32). This sample was again skewed young and male in comparison to the actual voting populations. Forty-nine percent of the participants indicated that they had a favorite candidate. The top two most frequently mentioned favorites were Sanders (*n* = 46) and Trump (*n* = 42).

#### Stimuli

Early in Spring 2019, we selected as stimuli potential US presidential candidates expressly running for a party’s nomination or mentioned in the media as possible candidates or vice-presidential running mates. Our final list consisted of 29 individuals: Louis Gutierrez, Stacey Abrams, Eric Holder, Cory Booker, Kamala Harris, Pete Buttigieg, Julian Castro, John Delaney, Tulsi Gabbard, Kirsten Gillibrand, Amy Klobuchar, Donald Trump, Bill Weld, Ted Cruz, Nikki Haley, Susana Martinez, Mike Pence, John Hickenlooper, Sherrod Brown, John Kasich, Mitt Romney, Beto O’Rourke, Elizabeth Warren, Bernie Sanders, Joe Biden, Wayne Messam, Bill de Blasio, Jay Inslee, and Howard Shultz. Good-quality YouTube videos of each candidate giving a formal speech while standing at a podium were downloaded and converted into 30-s thin slices using iMovie.

These thin slices were inserted into a Qualtrics survey alongside a 0- to 10-point, unipolar “thermometer” rating response tool (poles = *not at all* to *extremely*). Four surveys were created with one of four rating instructions: Please rate each leader for “receptivity (warmth and attractiveness)” or “formidability (power and competence)” or “charisma” or “authenticity.” Each survey began with a standard practice item to familiarize the perceivers with the response tool. Loading multiple 30-s videos can be slow and tedious, and so the survey was programmed to present random subsets of 20 of the 29 leaders to shorten the task and maintain engagement. The survey was distributed to participants *via* MTurk.

#### Procedure

The study was introduced to MTurk participants as “impressions of leaders.” No time limit was given for responding, and completion times varied between 18 and 44 min; on average, it took 24 min (*SD* = 16.79 min). The participants were randomly assigned to rate one of four traits. After responding to the online consent form, the participants viewed and rated subsets of leaders on the trait they were randomly assigned. The participants lastly reported their age, ethnicity, and gender.

### Results and Discussion

Political leader (*N* = 29) was the unit of analysis. Trait ratings were averaged across participants for each trait/leader combination (*n*s were between 42 and 54). These means formed the basis of each leader’s receptivity, formidability, authenticity, and charisma score.

Simultaneous regression analyses using charisma as the outcome variable and receptivity, formidability, and trustworthiness as predictors were run, with interaction terms included. Consistent with Hypothesis I, receptivity and formidability contributed independently and significantly to charisma; authenticity did not (see Table 3).

**TABLE 3:** Simultaneous regression analysis predicting the charisma of political leaders identified as potential candidates for the 2020 US presidential election (study 1c).

	*b*	*df*	*T*
Intercept	5.25	490.5	38.29
Receptivity	0.66	1,712.0	6.37***
Formidability	1.64	921.6	6.81***
Authenticity	–0.12	1,289	–0.70
Receptivity × formidability	–0.75	1,851	−3.38**

Leader was the unit of analysis (N = 29). *p < 0.05, **p < 0.01, ***p < 0.001.

**FIGURE 2:**
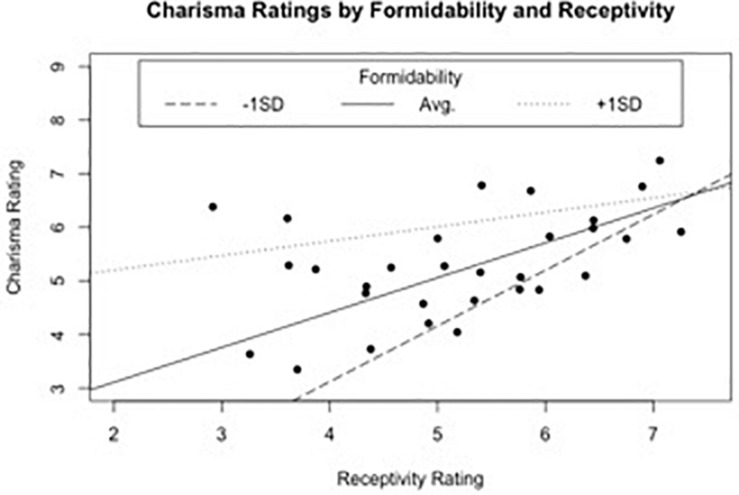
Depiction of the receptivity–formidability interaction for potential US candidates; receptivity scores had a greater impact on charisma at lower levels of formidability (study 1c).

Unlike the two previous studies, the interaction between receptivity and formidability was significant. We examined the interaction further by probing the receptivity–charisma relationship at low, average, and high levels of formidability. For this model, formidability was centered around + 1 SD from the mean (*M* = 5.56, *SD* = 0.52). The results for this analysis are depicted in Figure 2. The figure shows that while the slope for receptivity remained statistically significant, *b* = 0.27, *t*(24) = 2.26, *p* = 0.03, the relationship between receptivity and charisma was more strongly positive at lower levels of formidability and less strongly positive at higher levels of formidability.

Overall, studies 1a, 1b, and 1c support Hypotheses 1: impressions of leader receptivity and formidability independently contribute to perceptions of leader charisma.

## Study 1D: Perceptions of Jamaican Educational Leaders

The stimuli for studies 1a–c were mostly White and male. Would the same perceptual formula for charisma work for female leaders of color? Study 1d investigated perceptions of charisma in a sample of female leaders from the island nation of Jamaica.

A series of presentations by Jamaican educational leaders were chosen as stimuli. These women were selected because the camera work was of high quality and standardized for each speaker, and the setting was formal. Each woman presented remarks from a podium on a stage with a curtain as backdrop. Leader was the unit of analysis.

### Materials and Methods

#### Participants

A total of 414 eligible US voters (57.5% male) were recruited through MTurk to participate as perceivers in an online study. Their mean age was 36.3 years (*SD* = 10 years). The sample was identified as 72.9% White, 8.9% Black, 7.4% Asian American, 5.6% Hispanic, 2.9% Native American, and the rest as “other” or unidentified.

#### Stimuli

**FIGURE 3:**
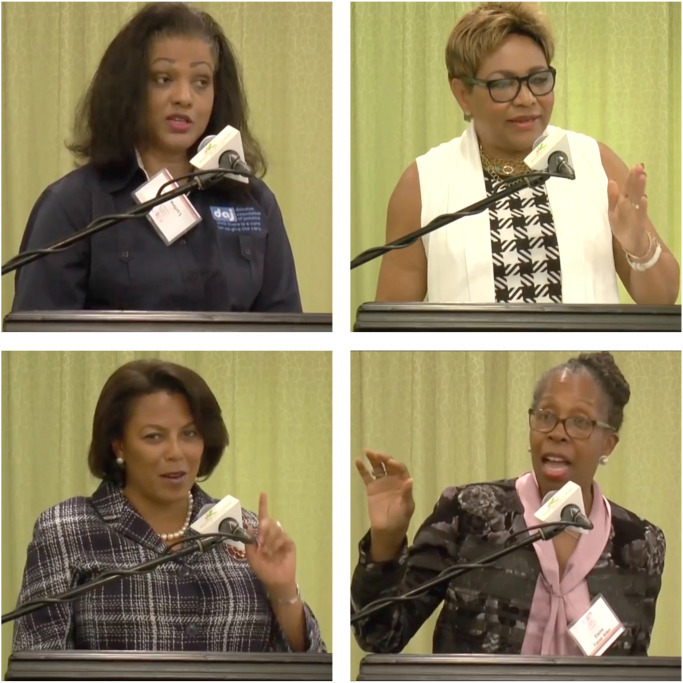
Still frames from a subset of Jamaican educational leaders’ thin slices used in study 1d.

Video stimuli were extracted from a publicly available YouTube channel of the Jamaican Teacher Association, which had posted the presentations of educational leaders at a recent conference in Jamaica. Presentations by 20 Black, female presenters were downloaded and edited using iMovie to create 30-s thin slices of each. Audio tracks, logos, titles, and other distractions were removed (see Figure 3 for examples).

The thin slices were inserted into a Qualtrics program and shared with participants through MTurk. Each thin slice was followed by a single, seven-point sliding scale (1 = not at all to 7 = very). As in study 1c, four surveys were created, instructing the participants to rate each leader for “receptivity (warmth and attractiveness)” or “formidability (power and competence)” or “charisma” or “trustworthiness.”

#### Procedure

The study was introduced to MTurk participants as “impressions of leaders” and took between 11 and 67 min to complete (*M* = 20 min). The participants were randomly assigned to rate one of four traits. After responding to the online consent form, the participants viewed and rated each of the 20 speakers (randomly presented) on the trait they were assigned. Afterward, the participants reported their age, ethnicity, and gender.

### Results and Discussion

Educational leader was the unit of analysis (*N* = 20). Ratings were averaged across participants to construct receptivity (*n* = 103), formidability (*n* = 99), trustworthiness (*n* = 112), and charisma (*n* = 100) scores for each leader.

Simultaneous regression analyses using charisma as the “outcome” variable and receptivity, formidability, and trustworthiness as “predictors” were initially run, with interaction terms included. Interactions were non-significant (*p*s > 0.15), and the results for the simplified analysis appear in Table 4. In support of Hypothesis I, receptivity and formidability contributed unique variance to impressions of charisma for Jamaican educational leaders; trustworthiness did not.

**TABLE 4:** Simultaneous regression analysis predicting female Jamaican educational leaders’ charisma (study 1d).

Trait	*b*	*t*
Receptivity	0.503	4.339***
Formidability	0.708	6.778***
Trustworthiness	–0.195	–1.713

Leader was the unit of analysis (N = 20). Scores for trait ratings were based on silent 30-s thin slices. For each trait rating, ns were receptivity = 103, formidability = 99, trustworthiness = 112, and charisma = 100. *p < 0.05, **p < 0.01, ***p < 0.001.

Thus, the findings for female Jamaican educational leaders are similar to those for political leaders in studies 1a and 1b, who were also not familiar to perceivers. These results contrasted with those for the recognizable US political leaders in study 1c which yielded the trait main effects plus their interaction. The consistent finding across all studies is that perceptions of charisma from nonverbal behavior depended upon receptivity and formidability displays.

## Study 2: Nonverbal Behaviors Related to Receptivity, Formidability, and Charisma

How do nonverbal signals and cues convey the dual-status messages of receptivity/formidability and project charisma? Our means of identifying behaviors potentially linked to these trait perceptions were largely exploratory and bottom-up. Our general hypothesis was that behaviors related to perceptions of receptivity and formidability would also be related to perceptions of charisma.

### Materials and Methods

#### Coding

Coding was based on a total of 68 political thin slices from studies 1a and 1b. Four additional clips from a pilot study conducted in Fall 2014 were also included. These showed speeches by Bob Menendez (D-NJ) and two featured Rand Paul (R-KY) (one domestic speech and one foreign speech each). The trait scores for these additional stimuli were acquired from a pool of 122 MTurk participants (in Fall 2014). Altogether these 68 thin slices represented our best controlled stimuli, as the setting (podium, background, audience) was relatively standardized (ten Brinke et al., 2015).

First, undergraduate research assistants content-analyzed thin slices of political speeches from studies 1a and 1b. They identified common face and body gestures and gaze patterns and constructed operational definitions. Next, new sets of trained undergraduate coders independently assessed each political speech for nonverbal behaviors. The coders were blind to the topic of the (muted) speeches and to the political leaders’ party and personal identity. The behaviors selected for exploration were clustered into three body areas: eyes and brows (looking up while speaking, blinking, brow frowns and raises), head and mouth (nods, shakes, smiles, frowns), and hand/arms (palms inward toward speaker, palms open toward audience, finger points, and fist-making). Gesture qualities were also coded, specifically the intensity of gesture (1–5 scale), counts of gestures outside and inside the body frame, and gestures directed toward or touching the body. Agreement between pairs of coders for each of the 16 coded behaviors was acceptable to excellent, ranging between Pearson *r* = 0.78 and 99. The details of our coding procedures appear in Supplementary Material for study 2.

### Results and Discussion

Video clip was the unit of analysis (*N* = 68). Correlations were computed between nonverbal behaviors and perceptions of receptivity, formidability, and charisma. Given the number of exploratory tests and the possibility of type I errors, alpha was set to *p* < 0.001, for a cumulative alpha of 0.05. Table 5 reports these results.

**TABLE 5:** Exploratory correlations between voter perceptions of political leaders’ receptivity, formidability, and charisma, and their nonverbal behaviors (study 2).

	Receptivity	Formidability	Charisma
**Eyes/brows**			
Look while speaking	0.28	0.27	0.40***
Blinks	–0.15	0.13	–0.02
Brow frowns	–0.03	–0.06	–0.05
Brow raises	0.28	–0.05	0.22
**Head/mouth**			
Nods	0.13	–0.04	0.11
Shakes	0.03	–0.00	0.06
Smiles	0.15	–0.13	0.10
Frowns	0.08	0.14	0.05
**Hand gestures**			
Palms to speaker	0.27	0.33	0.42***
Palms to audience	0.25	0.29	0.34
Points	0.18	0.13	0.21
Fists	0.01	0.11	–0.02
**Gesture intensity, expansiveness, direction**			
Intensity	0.36	0.49***	0.50***
Outside body frame	0.41***	0.38***	0.46***
Inside body frame	0.22	0.50***	0.45***
Toward the body	0.39***	0.21	0.34

Data were based on 30-s thin slices (the unit of analysis). Each political leader was sampled twice, giving two separate presentations (see text), N = 68. ***p < 0.001.

Overall, correlations emerging from measurements of the quality of gesturing (i.e., intensity, expansiveness, direction) were more in line with predictions than correlations for the gestures themselves. In particular, expansive gestures that ranged outside the frame of the body related to perceptions of all three traits. Impressions of formidability were significantly associated with both gesturing expansively and gesturing within the frame of the body and gesturing in an energetic, intense fashion. Bringing the hands toward the body signaled receptivity. Charisma related to looking at the audience while speaking and gesturing with palms toward the speaker. Nonverbal behaviors that showed little association with receptivity and formidability were generally uncorrelated with charisma (see Table 5), suggesting that impressions of receptivity and formidability gleaned from nonverbal signals are each essential to perceptions of charisma.

However, these data fail to capture the rich complexity of signaling. Nonverbal communication results from unfolding constellations of cues and signals (e.g., gaze plus point plus brow frown; shrug plus open palms plus smile), framed by expectations for sex, age, and status and contextualized by static cues (e.g., physiognomy, body build, and setting characteristics such as room size, podiums, and the distance, location, and other features of audiences). Behaviors present in different order combinations and with different qualities of action (e.g., intensity, direction, sequencing, repetition, speed, and rate of change), and the display of one action influences the likelihood of displaying another. Moreover, habits of the body are highly idiosyncratic, a fact that cartoonists and impressionists rely on to convey individual identity.

Had our coding been more fine-grained, we might have detected subtle distinctions in the elements and the qualities of gestures and gaze patterns and how they combined to influence meaning. Technologies enabling unobtrusive and precise recording and analysis of movements and expressions through time, aided by machine learning (Buck and Miller, 2016; Dael et al., 2016), are needed to help us understand how specific social signals are received, integrated, and processed in the brain (e.g., Church et al., 2016; Simon et al., 2020).

## Overview of Studies 3A and 3B: Charisma and Approach/Avoidance Motivation

Hypothesis III proposes that the receptivity/formidability displays of charismatic individuals have motivational consequences that distinguish them from non-charismatic individuals: they energize balanced, approach/avoidance motivation in perceivers. We tested this proposition at the level of brain systems using EEG to record neural activation in the left and the right brain hemispheres of perceivers as they watched thin slices of US and Jamaican educational leaders. Activation in the left frontal cortical region is associated with approach motivation, while activation in the right frontal cortical region is linked to avoidance motivation (Sutton and Davidson, 1997; Amodio et al., 2004; Boksem et al., 2012; Kelley et al., 2017). Relatively large discrepancies in alpha between hemispheres were predicted in response to non-charismatic leaders, thereby indicating either too much approach or too much avoidance activation. Relatively equal amounts of alpha activity in the left-frontal and right-frontal cortical areas were expected in response to viewing a charismatic leader. At the behavioral level, perceivers were expected to indicate on a four-option button box relatively greater consideration of voting for leaders high than low in charisma. Parallel predictions were made for non-charismatic and charismatic Jamaican educational leaders. In each study, undergraduate participants received lab experience credit as an incentive for participating.

## Study 3A

### Materials and Methods

#### Participants

The participants were 37 undergraduate students enrolled in introductory-level psychological science classes at a small liberal arts college in the northeastern US. Participants with > 50% poor channel recordings were omitted. This resulted in the exclusion of three participants’ data and a final *n* of 34 participants (19 females and 15 males). Twenty-eight participants were right-handed.

#### Stimuli

Selected 30-s thin slices of political leaders were used as stimuli. Results from the MTurk studies of US political leaders (studies 1a and 1b) were used to identify leaders perceived as high and low in charisma (i.e., more or less than 1 SD from the mean rating of charisma across all 68 leaders, respectively). Among the seven highly charismatic leaders were five Whites/two Blacks, four women/three men, and four Republicans/three Democrats. Among the group of seven scoring low in charisma were seven Whites, two women/five men, and four Republicans/three Democrats. These two subsets of thin slices comprised the high- and the low-charisma conditions for participants when they viewed political leaders. An additional thin slice of a White male politician who scored at the mean of charisma ratings was included as a practice item.

All 20 Jamaican leaders from study 1d were used as 30-s stimuli. Non-charismatic and charismatic Jamaican leaders were determined using a median split (< or > 4.39) on a seven-point rating scale for charisma. Thus, participants viewed 10 Jamaican leaders each in the low- and the high-charisma conditions.

#### Apparatus

Thirty-two channel EEGs were recorded in a sound-attenuated Faraday chamber using Electrical Geodesics Incorporated’s (EGI; Phillips Neuro) Geodesic EEG acquisition system (GES 400) with Geodesic Hydrocel sensor nets (electrolytic sponges). The online reference was at the vertex (Cz), and the impedances were maintained below 50 kΩ (EGI amplifiers are of high impedance). All EEG signals were amplified and sampled at 1,000 Hz.

Thirty-two channel nets were fitted on the participants’ heads using known anatomical landmarks. EEG was recorded from the frontal (Fp1, Fp2, F3, F4, F7, F8), central (C3, C4), temporal (T7, T8), parietal (P3, P4, P7, P8), and occipital (O1, O2) scalp regions, along the midline (Fz, Cz, Pz, Oz), and from 10 additional sites (Chatrian et al., 1985). Power in the alpha band (averaged across each 30-s thin slice) was recorded from separate, regionally homologous scalp electrodes in each hemisphere while the participant sat in a comfortable chair viewing the stimuli on the screen. The participants held a four-option button box to record how likely they would be to consider voting for the leader just observed (1 = not at all likely to 4 = very likely). A video camera installed in the chamber monitored the participant’s condition.

#### Procedure

The research was introduced as a study of impressions of leaders. The participants were told that they would view and rate silent video presentations of leaders. Each participant was given a brief explanation of the EEG recording procedure and was then prepared for physiological recording. First to appear was a practice thin slice (a White male politician of average charisma) to familiarize the participants with the task. The 14 political leaders’ thin slices were then presented, in random order, to each participant. After each stimulus presentation, the participants rated voting likelihood using the button box. A 2 min relaxation break occurred before the participants then viewed and rated each Jamaican stimulus in random order.

EEG data were preprocessed offline using NetStation 4.5 (Electrical Geodesic Inc., Eugene, OR, United States). The EEG data were first bandpass-filtered (0.3–100 Hz), bad channels were then identified and replaced, and an average reference was computed. The EEG signals were then exported to MATLAB for artifact rejection and power analysis. The clean data were then submitted to a Fast Fourier transform (FFT) using a 100% Hanning window.

### Results and Discussion

**FIGURE 4:**
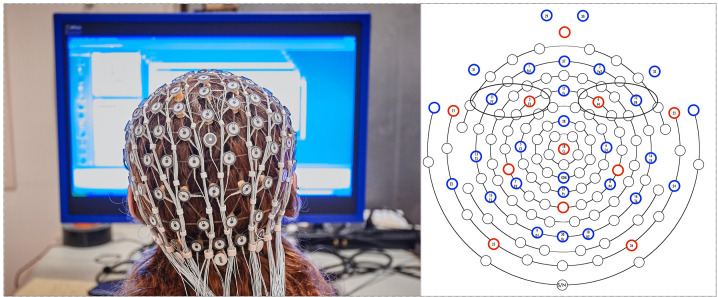
EEG 32 channel net and sensor map showing frontal electrode pairs F3–F4 and F7–F8 (circled) used to measure alpha in each hemisphere.

Based on findings from previous research (Boksem et al., 2012; Harmon-Jones and Gable, 2017), statistical analyses were confined to data measured from the electrode pairs F4/F3 and F8/F7 (see Figure 4). To obtain a measure of asymmetry in frontal brain activation, absolute difference scores were calculated by subtracting the spectral power value for the left and the right hemisphere electrodes, resulting in two measurements: the absolute difference of F4 minus F3 and the absolute difference of F8 minus F7. We predicted that non-charismatic leaders would stimulate uneven neural activity, reflecting either too much or too little approach and/or avoidance. Charismatic leaders were expected to activate similar amounts of neural activity in the alpha range in each frontal hemisphere.

**FIGURE 5:**
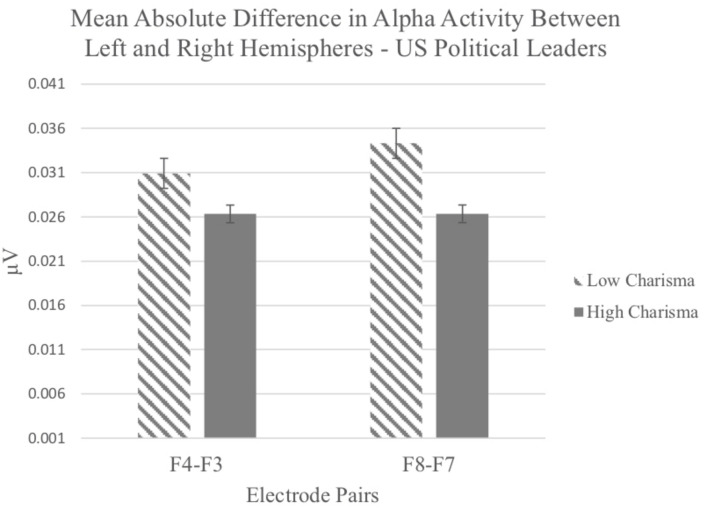
Mean absolute difference in alpha activity recorded from electrode pairs in perceiver’s left and right hemispheres in response to viewing US political leaders rated as high or low in charisma.

Data for the US political leaders and the Jamaican educational leaders were analyzed separately. For each analysis, we computed a two-way, repeated-measures analysis of variance (ANOVA) with charisma (low vs. high) and electrode pairs (F4–F3 vs. F8–F7) as factors. For US political leaders, the ANOVA indicated a statistically significant main effect of charisma, *F*(1, 33) = 9.006, *p* < 0.005, η^2^ = 0.214. As predicted, non-charismatic leaders produced greater differences in alpha across hemispheres relative to charismatic leaders, who generated more even amounts of signal from each hemisphere. There were no effects for electrode pair and no interaction (*p*s > 0.05) (see Figure 5).

**FIGURE 6:**
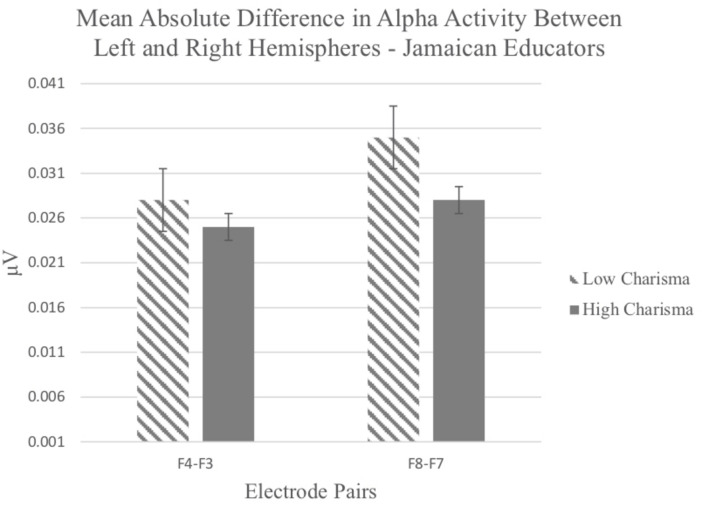
Mean absolute difference in alpha activity recorded from electrode pairs in perceiver’s left and right hemispheres in response to viewing Jamaican educational leaders rated as high or low in charisma.

Although means were in the predicted direction, the ANOVA for Jamaican educational leaders yielded no statistically significant main effect for charisma, *F*(1, 33) = 1.83, *p* = 0.155), and no other effects (see Figure 6).

Analyses of the voting intention data were consistent with predictions for both groups of leaders across the gender of participants: Leaders low in charisma generated less voting consideration. These analyses were framed as 2 (charisma: low/high) × 2 (participant gender) mixed-factor ANOVAs (with repeated-measures on the first factor) on the four-point vote intention scores. For US politicians, means of 2.08 (*SD* = 0.46) and 2.78 (*SD* = 0.37) emerged for low- and high-charisma leaders, respectively, *F*(1, 35) = 59.04, η^2^ = 0.628, *p* < 0.001. There was no main effect for gender of the participant and no interaction, *p*s > 0.75. For Jamaican educational leaders, means for low- and high-charisma leaders, 2.34 (*SD* = 0.36) and 2.72 (*SD* = 0.41), respectively, resulted in *F*(1, 35) = 52.77, η^2^ = 0.601, *p* < 0.001, with no main effect or interaction involving participant gender, *p*s > 0.21.

In sum, brain activity patterns in response to US politicians generated less hemisphere discrepancy in alpha power, supporting the contention that charisma balances approach and avoidance motivation (Hypothesis III). Comparable results for Jamaican leaders were not statistically reliable. Participant voting intention was stronger for charismatic than non-charismatic leaders across both sets of stimuli.

## Study 3B: Replication of Study 3A

Study 3b was designed as a replication of Study 3a using the same stimuli and a new set of participants. An additional, exploratory measure of approach/avoidance was added; the participants were instructed to lean forward or backward if the leader they watched made them feel like doing so. These responses were collected for a separate study not reported here.

### Materials and Methods

#### Participants

The participants were 50 undergraduate students (*M* = 19 years) enrolled in introductory-level psychological science classes at a small liberal arts college in the northeastern US. Due to an excessive number of poor channel recordings (>50%), eight participants’ data were excluded; so the analyses comprised 42 participants (27 females and 15 males). Thirty-six participants were right-handed. Unlike in study 3a, there was a 50% recognition rate for one stimulus, Cory Booker. These participants were maintained.

#### Stimuli

The stimuli were identical to those used in study 3a.

#### Apparatus

The basic equipment was identical to that used in study 3a except that an additional video camera was installed in the ceiling directly over the participant’s chair to track participant motion.

#### Procedure

Instructions were identical to those used in 3a except that the participants were asked to “lean forward or backward if the person in the video makes you feel you want to.” The participants were instructed on screen to return to a baseline seating position (i.e., to sit straight up) before the onset of each new stimulus.

The EEG data were preprocessed, exported to MATLAB for artifact rejection and power analysis, and submitted to FFT as in study 3a.

### Results and Discussion

Absolute difference scores for the key electrode pairs were computed as before for study 3a. Data for the US political leaders and the Jamaican educational leaders were analyzed separately as two-way, repeated-measures ANOVA, with charisma (low *vs.* high) and electrode pairs (F4–F3 vs. F8–F7) as factors. Overall, the results were similar to those for study 3a. For US political leaders, the ANOVA indicated a statistically significant main effect for charisma, *F*(1, 41) = 5.71, *p* < 0.022, η^2^ = 0.122. As predicted, non-charismatic leaders produced greater differences in alpha across hemispheres (*M* = 0.029, *SE* = 0.004) relative to charismatic leaders who generated more similar amounts of signal from each hemisphere (*M* = 0.023, *SE* = 0.003). There were no significant effects for electrode pair and no interaction (*p*s > 0.10).

Once again, although mean absolute differences in alpha between non-charismatic and charismatic were in the predicted direction (for non-charismatic, *M* = 0.026, *SE* = 0.004 and for charismatic, *M* = 0.023, *SE* = 0.003), the ANOVA for Jamaican leaders showed no statistically reliable main effect for charisma, *F*(1, 41) = 2.195, *p* = 0.146, η^2^ = 0.051 nor any other effects.

Consistent with study 3a, analyses of the voting intention data revealed relatively lower voting intention scores for leaders low in charisma. For US politicians, the 2 (charisma: low/high) × 2 (participant gender) mixed-factor ANOVA (repeated-measures on the first factor) on vote intention scores (four-point scale) yielded means of 1.99 (*SD* = 0.42) and 2.69 (*SD* = 0.39), respectively, *F*(1, 40) = 74.66, η^2^ = 0.65, *p* < 0.001. There was no main effect for gender of participant and no interaction, *p*s > 0.125. For Jamaican educational leaders, the comparable analysis yielded means of 2.39 (*SD* = 0.37) vs. 2.78 (*SD* = 0.45), for low- and high-charisma leaders, respectively, resulting in *F*(1, 40) = 29.66, η^2^ = 0.426, *p* < 0.001. There was no interaction involving participant gender (*p* > 0.21), but a main effect emerged, *F*(1, 40) = 13.52, η^2^ = 0.253, *p* < 0.001, indicating that females gave higher ratings overall to female Jamaican leaders than did males, *M* = 2.72, *SD* = 0.35 vs. *M* = 2.35, *SD* = 0.62.

In sum, there was evidence from study 3a and study 3b for US political leaders that charismatic displays tap approach and avoidance motivational systems in a roughly equal measure, consistent with Hypothesis III.

Although the alpha power patterns for Jamaican leaders were similar to those for US political leaders, they were not statistically significant. Among the likely reasons for the difference in outcomes was restriction of range in the measurement of Jamaican leader charisma. The US sample of leaders was triple the size of that for Jamaicans and selectively sampled to reflect extreme levels of charisma. The low and high charisma categories for Jamaicans rested on a median split of 20 individual scores. In addition, the Jamaican sample was culturally distinct and exclusively female. Larger diverse populations of real-world leaders *and* followers should be sampled to properly investigate cultural similarities and differences in the nonverbal formula for charismatic leadership.

## Study 4: Leader–Follower Relationships

Studies 1 through 3 offer evidence at the perceptual, behavioral, and brain systems level of analysis that charismatic leaders project the dual-status messages of receptivity and formidability *via* nonverbal signals and cues, thereby activating approach and avoidance motivation. Extending this thinking to the group level of analysis, hypothesis IV proposes that these psychological processes set conditions for the uniquely influential relationship charismatic leaders have with followers. Study 4 tests Hypothesis IV in undergraduate students, testing patterns for male and female followers separately.

In study 4, student observers evaluated established campus leaders given the task of persuading unfamiliar students to reach consensus in a group decision-making task. The leaders were video-recorded as they performed this task twice, once with female group members and once with male group members. Observers rated leaders for the expression of one of three traits: leader receptivity, formidability, or charisma. Leader influence was assessed using objective measures taken from the interactions (i.e., time to reach consensus, follower opinion changes toward the leader’s point of view). The predictions were that (1) perceptions of leader receptivity and formidability would contribute independently to perceptions of their charisma and (2) charismatic leaders would sway group member public and private opinion their way on a controversial decision and take less time to do it than would less charismatic leaders.

### Materials and Methods

#### Participants

For the main study, the participants who rated the leaders from thin slices (“observers”) were undergraduate students (48 females, 18 males, and one other) from a small liberal arts university in the northeast US. They received lab credit as an incentive. Observers ranged in age from 18 to 26 years (*M* = 19, *SD* = 1.36). The majority (84%) were White and middle to upper-middle class.

The participants instrumental to the creation of the stimuli consisted of campus leaders and *ad hoc* group members who attended the same university years earlier. The leaders were 40 undergraduate students (20 males/20 females). Each was offered the chance to earn up to $20 for their participation in a leadership study. The leaders were drawn from elected positions on campus. Collectively, they held leadership positions in a variety of organizations including fraternities, sororities, musical groups, athletic teams, religious organizations, student government, and musical groups. An additional 152 participants (74 men, 78 women) were recruited as *ad hoc* members of each leader’s group (“members”). The leaders were unfamiliar with the members. The members received laboratory credit for their participation. Problems with recordings or participant no-shows led to missing data on some variables, and one leader had to be dropped, reducing the total number of leaders to 39.

#### Stimuli

The stimuli used in study 4 originated from unpublished work on gender and leadership. The original videotapes of group interactions from this study were edited into 60-s thin slices (with 4-s inter-trial intervals), showing undergraduate leaders (*N* = 39) working to bring two undergraduate group members to consensus during a decision-making task. Both audio and visual information was maintained. The videos focused on the leader in the center of the shot as they interacted with two group members on either side of a square table. The group members were mostly out of view. Occasionally, a hand, arm, or partial profile of a group member would drift into the shot, but the facial and body reactions of group members were never evident in any of the videos.

The videos showed leaders working to gain group member consensus that a particular advertising storyboard (for gender-neutral products such as cereal, toothpaste, or fast food) was the most effective of four options. Each leader performed the task twice, once with two female group members and once with two male group members (order counterbalanced across leaders). Different sets of storyboards were presented to each male and female group to mitigate leader practice effects. Unbeknownst to group members, the leaders were incentivized to be persuasive by the promise of a cash reward for success, defined as the targeted ad being chosen by the group ($10 each round; in fact, all leaders were paid $20 regardless of outcome). To make consensus more difficult to achieve, group members (and leaders) individually pre-rated each storyboard for “effectiveness” before group interaction. Those ratings were averaged by the experimenters, and the second lowest-rated storyboard became the leaders’ target (though leaders believed the assignment was random).

These recordings were randomly separated into two sets of stimuli of between 34 and 38 thin slices, depending on missing recordings, so that observers saw only one of each leader’s two performances.

Objective measures of leader influence based on the decision-making task included (1) whether the group gave the targeted ad advocated by the leader its top rank (public opinion change) and (2) the time it took to reach consensus on the ad that earned top ranking. In addition, leader influence in changing members’ *private* opinions about the targeted ad was assessed. After discussion and having reached public consensus, the group members returned to individual cubicles and re-rated the original storyboards. Differences between pre-ratings and post-ratings in the direction of the ad the leader advocated for were summed up for each group member to indicate (3) private opinion change, the third measure of leader influence.

#### Procedure

Observers for the main study were run in a classroom in groups of 3–10. The study was introduced as an investigation of “first impressions of everyday leaders.” Observers signed consent forms and recorded their age, gender, and assigned ID number on the laptops provided to them. One randomly determined stimulus set was projected on a large screen in the front of the classroom so that the observers saw each leader only once. They used Qualtrics-presented one- to six-point sliding scales with poles marked “not at all” to “extremely” to rate each leader on one of three randomly assigned traits: receptivity [“indicate how receptive (warm, attractive) you perceive the leader to be”], formidability [“indicate how formidable (powerful, competent) you perceive the leader to be”], or charismatic [“indicate how charismatic you perceive the leader to be”).

The task took 50 minutes. To make sure that observers were kept engaged, three 2 min breaks occurred. During these pauses, the observers were told to sit back, close their eyes, and relax and that no electronics could be used.

### Results and Discussion

The subgroups of observers were consistent in their judgments of the three traits from the thin-sliced video and audio information. Intraclass correlation coefficients ranged from acceptable too strong for receptivity, formidability, and charisma, respectively, for set 1 = 0.70 (*n* = 9), 0.92 (*n* = 12), 0.93 (*n* = 12), and for set 2 = 0.63 (*n* = 11), 0.86 (*n* = 11), and 0.94 (*n* = 11).

The unit of analysis was leader. Impressions of leader charisma, separately for male and female member groups, were simultaneously regressed on perceptions of receptivity and formidability. The receptivity/formidability interaction was entered next. The addition of the interaction term to the model added no significant explanatory power for leader performances with either male groups, *R*^2^ change = 0.001, *F*(1, 31) < 1, or female groups, *R*^2^ change = 0.001, *F*(1, 34) < 1. Therefore, each interaction term was dropped. Table 6 shows the results of the simple models. Whether thin slices captured leader interactions with male or female group members, perceptions of leader charisma by external peer observers were predicted by other peer observers’ perceptions of leader receptivity and leader formidability.

**TABLE 6:** Simultaneous regression analyses predicting student leaders’ charisma from independent observer ratings of leader receptivity and formidability (study 4).

		Receptivity		Formidability	
	*R*^2^	*b*	*t*	*b*	*t*
**Interaction pairs**					
Male	0.84	0.39	4.40***	0.63	7.22***
Female	0.87	0.55	6.83***	0.47	5.78***

Perceptions were based on 60-s thin slices of student leaders (N = 39) shown interacting with unique pairs of male and female followers. *p < 0.05, **p < 0.01, ***p < 0.001.

Next, leader charisma was correlated with each measure of leader influence. The average time to come to consensus for leaders interacting with males was 7.75 min and for females was 5.85 min. When leaders interacted with male group members (*n* = 37), neither the achievement of publicly expressed consensus on the ad, *r* = −0.06, nor the time it took to come to some consensus, *r* = 0.077, related to raters’ perceptions of leader charisma. However, charisma and private opinion change in the direction of leaders’ persuasive messages was positively associated, *r* = 0.334, *p* < 0.05. When leaders interacted with female group members (*n* = 39), both public consensus, *r* = 0.32, *p* < 0.05, and private opinion change, *r* = 0.42, *p* < 0.01, were positively associated with charisma; again, time to reach consensus was not, *r* = −0.21, *p* > 0.05.

Study 4 offers partial support for Hypothesis IV. As predicted, assessments of perceived receptivity and formidability made during the first 60 s of group interaction by independent samples of observers predicted other observers’ perceptions of leader charisma. Results for leader influence were mixed as leader charisma predicted only some aspects of eventual leader potency. In line with predictions, female and male group members’ private opinions changed in the direction of the leaders’ advocated point-of-view assessed after group interaction. This is consistent with the idea that followers of charismatic leaders develop unusual degrees of dependency on them (Kark et al., 2003). However, leader charisma predicted public consensus only when group members were female. In addition, leader charisma was unrelated to the time it took to reach consensus, contrary to predictions.

## General Discussion

How do single individuals come to influence the beliefs and the actions of many? This is arguably the central mystery of charisma as it relates to leadership. Leadership scholars have made the case for charisma’s evolutionary roots (e.g., Van Vugt et al., 2008; Day and Antonakis, 2012; Castelnovo et al., 2017; Grabo et al., 2017), and researchers have identified some of its crucial, nonverbal processes and components (e.g., Erez et al., 2008; Riggio and Riggio, 2010; Reh et al., 2017; Tskhay et al., 2017; Sy et al., 2018). Crossing levels of analysis, we attempted to fill gaps in explanation based on status cues theory, the idea that nonverbal status cues drive perceptions, motivations, behaviors, and relationships. We hypothesized and found qualified, preliminary evidence that charismatic, nonverbal displays lend impressions of receptivity and formidability, stimulate perceivers’ approach/avoidance motivations in relatively equal measure, and set conditions for uniquely potent psychological influence.

Discovering that charisma’s perceptual foundations lie in the dual-status messages of receptivity and formidability is not surprising, given the well-established dimensions of attractiveness/warmth and agency/power/competence in the general social cognition literature (Rosenberg et al., 1968; Judd et al., 2005; Fiske et al., 2007). More interesting was the fact that these status messages rarely interacted with one another, which means that, in most of the studies reported here, their dual impacts on leader charisma were additive rather than compensatory or synergistic. Consistent with status cues theory, each attribution contributed to charisma independently of the other, presumably activating both approach and avoidance motivational systems. In the future, researchers should measure the live action sequencing of charismatic leader displays to see how motivational brain systems are harnessed to make followers more susceptible to influence (Deng et al., 2018; Schnuerch and Pfattheicher, 2018).

Where perceptions of receptivity and formidability did moderate each other was among the 29 recognizable political leaders in study 1c. While each trait contributed to perceived charisma, the association between receptivity and charisma was more strongly positive at lower levels of formidability than at higher levels. It is tempting to speculate that this result somehow reflects the prevalence of anger displays by two of the top candidates in our pool (Trump and Sanders). More directly, it brings to light a distinction embedded within the perceptual and the motivational levels of the status cues approach. Perceptually, anger appears formidable and non-receptive, but motivationally, anger is processed as an approach emotion and stimulant for the left hemisphere (Harmon-Jones and Sigelman, 2001; Carver and Harmon-Jones, 2009). Thus, balanced approach/avoidance motivation may sometimes be achieved through the “attractant” of anger signaling which register on the perceptual scale as formidable.

There are additional nuances in our data that warrant future exploration. In the eyes of potential voters who made attributions from leader thin slices, the explanatory power of receptivity and formidability in the nonverbal formula for charisma did not vary across the accessible cue of leader gender. Though voters were unaware of leader party and heard no speech content, these variables moderated how charisma was perceived (see Supplementary Material for studies 1a and b). Specifically, the receptivity–charisma link was stronger for Democratic than Republican leaders and stronger for all leaders when they spoke about domestic compared to foreign issues. Consciously or non-consciously, charismatic Democratic leaders perhaps exude a “kinder, gentler” nonverbal self compared with charismatic Republicans, and charismatic leaders from both parties may do the same when speaking about domestic concerns vs. foreign affairs. These possibilities crosscut studies reporting more perceived warmth in the physiognomies of Democratic compared to Republican elected officials (Rule and Ambady, 2010) and preferences for feminine-looking leaders when crises involve ingroup social welfare rather than intergroup competition (e.g., Spisak et al., 2012).

Perceptions of trustworthiness and authenticity were tested as compelling alternatives to receptivity and formidability signals (e.g., Todorov et al., 2008; Van‘t Wout and Sanfey, 2008; Rule et al., 2013). However, they showed weak and/or inconsistent relationships with charisma relative to the explanatory power receptivity and formidability offered. This pattern held true whether leaders were unrecognized (studies 1a, 1b, and 1d) or whether they were candidates on the campaign trail, verging on celebrity status (study 1c).

The sampling of real-world leaders offers cautionary tales and insights about the role of charisma in politics. First, charisma ratings were undoubtedly affected by the quality of visual framing in selected clips (Stewart et al., 2018). This potential confound was a trade-off to using stimuli with high, real-world validity. Future studies should deploy advanced digital editing/machine learning techniques. Second, charisma measured by thin slice had limited predictive power with regard to political fortunes. The potential 2020 election candidates that we studied in Spring 2019 with the highest charisma raw scores were, in order, Booker, Gutierrez, Harris, O’Rourke, Trump, Sanders, and Klobuchar. All but Harris and Trump fell by the campaign trail wayside. Biden, the Democratic party’s presidential nominee, was not perceived as especially charismatic. Notably, the top three scorers were people of color.

In fact, though our three US samples of political leaders were skewed male and White, people of color were disproportionally perceived as highly charismatic. In particular, the highly charismatic US leaders selected as stimuli for the EEG studies (studies 3a and 3b) comprised more political leaders of color and women than did the subset of leaders selected as exceptionally low in charisma (who were predominantly White and male). Race and/or gender may have introduced systematic error in our results. Still, why did political leaders of color appear disproportionally charismatic in our samples? Because of racial biases, perhaps only the most charismatic communicators of color gain entry into the White-male-dominated political institutions of government. White males, on the other hand, may have less developed communication skills because they are able to access positions in government from multiple points of entry.

The status messages that charismatic performances draw upon are rooted in the evolution of human gestural and morphological communication systems designed to regulate the foundational motivations of social life: approach and avoidance. Charismatic individuals capture us by using some of the oldest tools on the planet, exuding receptivity and formidability in ways we experience as mixed motivations to approach and withdraw. It may be in our nature to succumb to their influence: devotion can be deeply cultivated when the object of attachment and the agent of threat are one and the same (Keating et al., 2005). Looking through the lens of status cues offers a view of the processes by which charismatic leaders with good and bad intentions motivate followers to believe, follow, act, and sacrifice.

## Data Availability Statement

The datasets generated for this study are available on request to the corresponding author.

## Ethics Statement

The studies involving human participants were reviewed and approved by the Colgate University Institutional Review Board. The patients/participants provided their written informed consent to participate in this study.

## Author Contributions

FA: studies 1d and 3a. HL: study 1a. WS: study 1b. KA: study 3. JH: study 1c. All authors listed helped in designing, writing the ethics proposal, ran, analyzed and wrote at least one or more of the multiple studies involved.

## Conflict of Interest

The authors declare that the research was conducted in the absence of any commercial or financial relationships that could be construed as a potential conflict of interest.

## Footnotes

1 Status cues theory considers both nonverbal cues and signals as potential transmitters of status information, sometimes conveyed intentionally and sometimes not (see Keating, 1985, 2002; cf. Laidre and Johnstone, 2013).

2 Some argue that the art of the deal between the closeness and the distance leaders maintain with constituents has been mastered by President Trump through his use of Twitter and personal phone calls (Hulse, 2019).

3 The first 100 words of two different opinion articles, one designed to invoke thoughts of external threat (terrorism on US soil) and the other designed to stimulate thoughts of internal threat (income inequality), were inserted into the Qualtrics survey. Four true/false questions pertaining to the subject matter of each opinion piece were used to check that participants read and understood the gist of the article they were presented with. Analyses showed that, though the participants read the primes, they had no impact on subsequent ratings. The data presented here are collapsed across prime.

## References

[B1] Adams R. B. Jr Nelson A. J. (2016). “Eye behavior and gaze,” in *APA Handbook of Nonverbal Communication*, eds Matsumoto D. (Washington, DC: American Psychological Association), 335–362.

[B2] Amodio D. M. Shah J. Y. Sigelman J. Brazy P. C. Harmon-Jones E. (2004). Implicit regulatory focus associated with asymmetrical frontal cortical activity. *J. Exp. Soc. Psychol.* 40 225–232. 10.1016/S0022-1031(03)00100-8

[B3] Anderson C. Kilduff G. J. (2009). Why do dominant personalities attain influence in face-to-face groups? The competence-signaling effects of trait dominance. *J. Pers. Soc. Psychol.* 96 491–503. 10.1037/a0014201 19159145

[B4] Antonakis J. Bastardoz N. Jacquart P. Shamir B. (2016). Charisma: an ill-defined and ill-measured gift. *Annu. Rev. Org. Psychol. Org. Behav.* 3 293–319.

[B5] Antonakis J. Jacquart P. (2013). *The Far Side of Leadership: Rather Difficult to Face. Exploring Distance in Leader– Follower Relationships: When Near is Far and Far is Near.* Milton Park: Taylor & Francis Group, 155–187.

[B6] Baron R. S. (2000). Arousal, capacity, and intense indoctrination. *Personal. Soc. Psychol. Rev.* 4 238–254. 10.1207/s15327957pspr0403_3

[B7] Bligh M. C. Riggio R. (2013). *When Near is Far and Far is Near: Exploring Distance in Leader–Follower Relationships.* New York, NY: Routledge.

[B8] Boksem M. Smolders R. De Cremer D. (2012). Social power and approach-related neural activity. *Soc. Cogn. Affect. Neurosci.* 7 516–520. 10.1093/scan/nsp006 19304842 PMC3375884

[B9] Bowlby J. (1982). *Attachment and Loss.* New York, NY: Basic Books.

[B10] Buck R. Miller M. (2016). “Measuring the dynamic stream of display: Spontaneous and intentional facial expression and communication,” in *APA Handbook of Nonverbal Communication*, eds Matsumoto D. Hwang H. C. Frank M. G. (Washington, DC: APA), 425–458. 10.1037/14669-017

[B11] Buck R. Renfro Power S. (2006). “The biological foundations of social organization: The dynamic emergence of social structure through nonverbal communication,” in *The Sage Handbook of Nonverbal Communication*, eds Manusov V. Patterson M. L. (Thousand Oaks, CA: Sage), 119–138. 10.4135/9781412976152.n7

[B12] Buhrmester M. Kwang T. Gosling S. D. (2011). Amazon’s mechanical turk: a new source of inexpensive, yet high-quality, data? *Perspect. Psychol. Sci.* 6 3–5. 10.1177/1745691610393980 26162106

[B13] Cairns R. B. (1979). *Social Development: The Origins and Plasticity of Interchanges.* San Francisco, CL: Freeman.

[B14] Carver C. S. Harmon-Jones E. (2009). Anger is an approach-related affect: evidence and implications. *Psychological Bulletin* 135 183–204. 10.1037/a0013965 19254075

[B15] Castelnovo O. Popper M. Koren D. (2017). The innate code of charisma. *Leadersh. Quart.* 28 543–554. 10.1016/j.leaqua.2016.11.003

[B16] Chatrian G. E. Lettich E. Nelson P. L. (1985). Ten percent electrode system for topographic studies of spontaneous and evoked EEG activities. *Am. J. EEG Technol.* 25 83–92. 10.1080/00029238.1985.11080163

[B17] Cherulnik P. D. Donely K. A. Wiewel T. S. R. Miller S. R. (2001). Charisma is contagious: the effect of leaders’ charisma on observers’ affect. *J. Appl. Soc. Psychol.* 31 2149–2159. 10.1111/j.1559-1816.2001.tb00167.x

[B18] Church R. B. Kelly S. D. Wakefield E. (2016). “Measuring gesture,” in *APA Handbook of Nonverbal Communication*, eds Matsumoto D. Hwang H. C. Frank M. G. (Washington, DC: APA), 499–524. 10.1037/14669-019

[B19] Crump M. J. McDonnell J. V. Gureckis T. M. (2013). Evaluating amazon’s mechanical turk as a tool for experimental behavioral research. *PLoS One* 8:14. 10.1371/journal.pone.0057410 23516406 PMC3596391

[B20] Dael N. Bianhi-Berthouze N. Kleinsmith A. Mohr C. (2016). “Measuring body movement: Current and future directions in proxemics and kinesics,” in *APA Handbook of Nonverbal Communication*, eds Matsumoto D. Hwang H. C. Frank M. G. (Washington, DC: APA), 551–588. 10.1037/14669-022

[B21] Dawkins R. Krebs J. R. (1978). “Animal signals: Information or manipulation?,” in *Behavioural Ecology: An Evolutionary Approach*, Vol. 2 eds Krebs J. R. Davies N. B. (Sunderland, MA: Sinauer Associates), 282–312.

[B22] Day D. V. Antonakis J. (2012). Leadership: past, present, and future. *Nat. Leadersh.* 2 3–25.

[B23] Delton A. W. Sell A. (2014). The co-evolution of concepts and motivation. *Curr. Dir. Psychol. Sci.* 23 115–120. 10.1177/0963721414521631 25221389 PMC4159186

[B24] Deng M. Zheng M. Guinote A. (2018). When does power trigger approach motivation? Threats and the role of perceived control in the power domain. *Soc. Personal. Psychol. Compass.* 12:e12390 10.1111/spc3.12390

[B25] Eagly A. H. Johannesen-Schmidt M. C. van Engen M. L. (2003). Transformational, transactional, and laissez-faire leadership styles: a meta-analysis comparing women and men. *Psychol. Bull.* 129 569–591. 10.1037/0033-2909.129.4.569 12848221

[B26] Erez A. Misangyi V. F. Johnson D. E. LePine M. A. Halverson K. C. (2008). Stirring the hearts of followers: charismatic leadership as the transferal of affect. *J. Appl. Psychol.* 93 602–615. 10.1037/0021-9010.93.3.602 18457489

[B27] Fiske S. T. Cuddy A. J. Glick P. (2007). Universal dimensions of social cognition: warmth and competence. *Trends Cogn. Sci.* 11 77–83. 10.1016/j.tics.2006.11.005 17188552

[B28] Galanter M. (1999). *Cults: Faith, Healing, and Coercion*, 2nd Edn New York, NY: Oxford University Press.

[B29] Goleman D. Boyatzis R. McKee A. (2000). *Primal Leadership: Realizing the Power of Emotional Intelligence.* Brighton, MA: Harvard Business School Press.

[B30] Grabo A. Spisak B. R. van Vugt M. (2017). Charisma as signal: an evolutionary perspective on charismatic leadership. *Leadersh. Quart.* 28 473–485. 10.1016/j.leaqua.2017.05.001

[B31] Gray J. H. Densten I. L. (2007). How leaders woo followers in the romance of leadership. *Appl. Psychol.* 56 558–581. 10.1111/j.1464-0597.2007.00304.x

[B32] Harlow H. H. (1959). Love in infant monkeys. *Sci. Am.* 200 68–74. 10.1038/scientificamerican0659-68 13658993

[B33] Harmon-Jones E. Gable P. A. (2017). On the role of asymmetric frontal cortical activity in approach and withdrawal motivation: an updated review of the evidence. *Psychophysiology* 55:e12879. 10.1111/psyp.12879 28459501

[B34] Harmon-Jones E. Gable P. A. Peterson C. K. (2010). The role of asymmetric frontal cortical activity in emotion-related phenomena: a review and update. *Biol. Psychol.* 84 451–462. 10.1016/j.biopsycho.2009.08.010 19733618

[B35] Harmon-Jones E. Sigelman J. (2001). State anger and prefrontal brain activity: evidence that insult- related relative left-prefrontal activation is associated with experienced anger and aggression. *J. Personal. Soc. Psychol.* 80 797–803. 10.1037/0022-3514.80.5.797 11374750

[B36] Haslam S. A. Reicher S. D. Platow M. J. (2010). *The New Psychology of Leadership: Identity, Influence, and Power.* New York, NY: Psychology Press.

[B37] Hertenstein M. J. (2011). “The communicative functions of touch in adulthood,” in *The Handbook of Touch: Neuroscience, Behavioral, and Health Perspectives*, eds Hertenstein M. J. Weiss S. J. (New York: Springer), 299–327.

[B38] Hogg M. A. (2001). A social identity theory of leadership. *Personal. Soc. Psychol. Rev.* 5 184–200. 10.1207/s15327957pspr0503_1

[B39] Hogg M. A. (2014). From uncertainty to extremism: social categorization and identity processes. *Curr. Dir. Psychol. Sci.* 23 338–342. 10.1177/0963721414540168

[B40] Howell J. M. Shamir B. (2005). The role of followers in the charismatic leadership process: relationships and their consequences. *Acad. Manag. Rev.* 30 96–112. 10.5465/amr.2005.15281435

[B41] Hulse C. (2019). *The President Is on the Line, and That Helps Keep the G.O.P. in Line.* New York, NY: New York Times.

[B42] Judd C. M. James-Hawkins L. Yzerbyt V. Kashima Y. (2005). Fundamental dimensions of social judgment: understanding the relations between judgments of competence and warmth. *J. Personal. Soc. Psychol.* 89 899–913. 10.1037/0022-3514.89.6.899 16393023

[B43] Kark R. Shamir B. Chen G. (2003). The two faces of transformational leadership: empowerment and dependency. *J. Appl. Psychol.* 88 246–255. 10.1037/0021-9010.88.2.246 12731708

[B44] Keating C. F. (1985). “Human dominance signals: The primate in us,” in *Power, Dominance, and Nonverbal Behavior*, eds. Ellyson S. Dovidio J. F. (New York, NY: Springer), 89–108.

[B45] Keating C. F. (2002). “Charismatic faces: Social status cues put face appeal in context,” in *Advances in visual cognition Facial Attractiveness*, Vol. I, eds Rhodes G. Zebrowitz L. A. (Westport, CT: Ablex), 153–192.

[B46] Keating C. F. (2011). “Channeling charisma through face and body status cues,” in *Social Psychological Dynamics*, eds Chadee D. Kostic A. (Jamaica: University of the West Indies Press), 93–111.

[B47] Keating C. F. (2018). “About face! Facial status cues and perceptions of charismatic leadership,” in *The Facial Displays of Leaders*, ed. Senior C. (London: Palgrave).

[B48] Keating C. F. Doyle J. (2002). The faces of desirable mate and dates contain mixed social status cues. *J. Exp. Soc. Psychol.* 38 414–424. 10.1016/s0022-1031(02)00007-0

[B49] Keating C. F. Pomerantz J. Pommer S. D. Ritt S. J. Miller L. M. McCormick J. (2005). Going to college and unpacking hazing: a functional approach to decrypting initiation practices among undergraduates. *Group Dyn.* 9 104–126. 10.1037/1089-2699.9.2.104

[B50] Kelley N. J. Hortensius R. Schutter D. J. L. G. Harmon-Jones E. (2017). The relationship of approach/avoidance motivation and asymmetric frontal cortical activity: a review of studies manipulating frontal asymmetry. *Int. J. Psychophysiol.* 119 19–30. 10.1016/j.ijpsycho.2017.03.001 28288803

[B51] Klimesch W. Doppelmayr M. Hanslmayr S. (2006). Upper alpha ERD and absolute power: their meaning for memory performance. *Prog. Brain Res.* 159 151–165. 10.1016/S0079-6123(06)59010-7 17071229

[B52] Knowles K. K. (2018). “The evolutionary psychology of leadership trait perception,” in *The Facial Displays of Leaders*, ed. Senior C. (Palgrave), 97–122. 10.1007/978-3-319-94535-4_5

[B53] Krieglmeyer R. Deutsch R. De Houwer J. De Raedt R. (2010). Being moved: valence activates approach-avoidance behavior independently of evaluation and approach-avoidance intentions. *Psychol. Sci.* 21 607–613. 10.1177/0956797610365131 20424109

[B54] Laidre M. E. Johnstone R. A. (2013). Animal signals. *Curr. Biol.* 23 829–833. 10.1016/j.cub.2013.07.070 24070440

[B55] Liberman N. Trope Y. (2008). The psychology of transcending the here and now. *Science* 322 1201–1205. 10.1126/science.1161958 19023074 PMC2643344

[B56] Lukaszewski A. W. Simmons Z. L. Anderson C. Roney J. R. (2016). The role of physical formidability in human social status allocation. *J. Personal. Soc. Psychol.* 110 385–406. 10.1037/pspi0000042 26653896

[B57] Mazur A. C. (1985). A biosocial model of status in face-to-face primate groups. *Soc. Forces* 64 377–402. 10.1093/sf/64.2.377

[B58] Mazur A. C. (2005). *Biosociology of dominance and deference.* New York, NY: Rowman & Littlefield Publishers.

[B59] Moore M. M. (2010). Human nonverbal courtship behavior: a brief historical review. *J. Sex Res.* 47 171–180. 10.1080/00224490903402520 20358459

[B60] Pastor J. C. Mayo M. Shamir B. (2007). Adding fuel to fire: the impact of followers’ arousal on ratings of charisma. *J. Appl. Psychol.* 92 1584–1596. 10.1037/0021-9010.92.6.1584 18020798

[B61] Rajecki D. W. Lamb M. E. Obmascher P. (1978). Toward a general theory of infantile attachment: a comparative review of aspects of the social bond. *Behav. Brain Sci.* 1 417–436. 10.1017/s0140525x00075816

[B62] Razin M. A. Kark R. (2012). “The apple does not fall far from the tree: Steve Jobs’s leadership as simultaneously distant and close,” in *Exploring Distance in Leader-Follower Relationship: When Near is Far and Far is Near*, eds Bligh M. C. Riggio R. E. (New York: Routledge), 241–273.

[B63] Reh S. Van Quaquebeke N. Giessner S. R. (2017). The aura of charisma: a review on the embodiment perspective as signaling. *Leadersh. Quart.* 28 486–507. 10.1016/j.leaqua.2017.01.001

[B64] Riggio H. R. Riggio R. E. (2010). Appearance-based trait inferences and voting: evolutionary roots and implications for leadership. *J. Nonverb. Behav.* 34 119–125. 10.1007/s10919-009-0083-0

[B65] Riggio R. E. Tan S. J. (eds) (2013). *Leader Interpersonal and Influence Skills.* New York, NY: Routledge.

[B66] Rosenberg S. Nelson C. Vivekananthan P. S. (1968). A multidimensional approach to the structure of personality impressions. *J. Personal. Soc. Psychol.* 9 283–294. 10.1037/h0026086 5670821

[B67] Rule N. O. Ambady N. (2010). Democrats and Republicans can be differentiated from their faces. *PLoS One* 5:e8733. 10.1371/journal.pone.0008733 20090906 PMC2807452

[B68] Rule N. O. Krendl A. C. Ivcevic Z. Ambady N. (2013). Accuracy and consensus in judgments of trustworthiness from faces: behavioral and neural correlates. *J. Personal. Soc. Psychol.* 104 409–426. 10.1037/a0031050 23276271

[B69] Schaller M. (2008). “Evolutionary bases of first impressions,” in *First Impressions*, eds Ambady N. Schaller J. J. (New York, NY: Guilford Press), 15–34.

[B70] Schnuerch R. Pfattheicher S. (2018). Motivated malleability: frontal cortical asymmetry predicts the susceptibility to social influence. *Soc. Neurosci.* 13 480–494. 10.1080/17470919.2017.1355333 28699831

[B71] Shamir B. (1995). Social distance and charisma: theoretical notes and an exploratory study. *Leadersh. Quart.* 6 19–47. 10.1016/1048-9843(95)90003-9

[B72] Shamir B. (2013). “Notes on distance and leadership,” in *Exploring Distance in Leader-Follower Relationship: When Near is Far and Far is Near*, eds Bligh M. C. Riggio R. E. (New York, NY: Routledge), 241–273.

[B73] Shamir B. House R. J. Arthur M. B. (1993). The motivational effects of charismatic leadership: a self-concept based theory. *Org. Sci.* 4 577–594. 10.1287/orsc.4.4.577 19642375

[B74] Simon J. C. Styczynski N. Gutsell J. N. (2020). Social perceptions of warmth and competence influence behavioral intentions and neural processing. *Cogn. Affect. Behav. Neurosci.* 20 265–275. 10.3758/s13415-019-00767-3 31965474 PMC7220095

[B75] Spezio M. L. Rangel A. Alvarez R. M. O’Doherty J. P. L. Mattes K. (2008). A neural basis for the effect of candidate appearance on election outcomes. *SCAN* 3 344–352. 10.1093/scan/nsn040 19015087 PMC2607056

[B76] Spisak B. R. Homan A. C. Grabo A. Van Vugt M. (2012). Facing the situation: testing a biosocial contingeny model of leadership in intergroup relationships using masculine and feminine faces. *Leadersh. Quart.* 23 273–280. 10.1016/j.leaqua.2011.08.006

[B77] Stewart P. A. Svetieva, El, Eubanks A. Miller J. M. (2018). “Facing your competition: findings for the 2016 Presidential election,” in *The Facial Displays of Leaders*, ed. Senior C. (London: Palgrave), 51–72. 10.1007/978-3-319-94535-4_3

[B78] Strack F. Deutsch R. (2004). Reflective and impulsive determinants of social behavior. *Personal. Soc. Psychol. Rev.* 8 220–247. 10.1207/s15327957pspr0803_1 15454347

[B79] Sutton S. K. Davidson R. J. (1997). Prefrontal brain asymmetry: a biological substrate of the behavioral approach and inhibition systems. *Psychol. Sci.* 8 204–210. 10.1111/j.1467-9280.1997.tb00413.x

[B80] Sy T. Horton C. Riggio R. (2018). Charismatic leadership: eliciting and channeling follower emotions. *Leadersh. Quart.* 29 58–69. 10.1016/j.leaqua.2017.12.008

[B81] ten Brinke L. Liu C. C. Keltner D. Srivastava S. B. (2015). Virtues, vices, and political influences in the U.S. *Senate*. *Psychol. Sci.* 27 85-93. 26581946 10.1177/0956797615611922

[B82] Todorov A. Baron S. G. Oosterhof N. N. (2008). Evaluating face trustworthiness: a model based approach. *Soc. Cogn. Affect. Neurosci.* 3 119–127. 10.1093/scan/nsn009 19015102 PMC2555464

[B83] Tskhay K. O. Zhu R. Zou C. Rule N. O. (2017). Charisma in everyday life: conceptualization and validation of the general charisma inventory. *J. Personal. Soc. Psychol.* 114 131–152. 10.1037/pspp0000159 28737418

[B84] Vacharkulksemsuk T. Reit E. Khambatta P. Eastwick P. W. Finkel E. J. Carney D. R. (2016). Dominant, open nonverbal displays are attractive at zero-acquaintance. *PNAS* 113 4009–4014. 10.1073/pnas.1508932113 27035937 PMC4839399

[B85] Van Vugt M. Hogan R. Kaiser R. B. (2008). Leadership, followership, and evolution: some lessons from the past. *Am. Psychol.* 63 182–196. 10.1037/0003-066x.63.3.182 18377108

[B86] Van‘t Wout M. Sanfey A. G. (2008). Friend or foe: the effect of implicit trustworthiness judgments in social decision-making. *Cognition* 108 796–803. 10.1016/j.cognition.2008.07.002 18721917

[B87] Wiltermuth S. S. Heath C. (2008). Synchrony and cooperation. *Psychol. Sci.* 20:115. 10.1111/j.1467-9280.2008.02253.x 19152536

