# Supplementary material for: Charismatic Nonverbal Displays by Leaders Signal Receptivity and Formidability, and Tap Approach and Avoidance Motivational Systems
Source: Front Psychol. 2020 Oct 22;11:526288. doi: 10.3389/fpsyg.2020.526288 (PMC7643026; doi:10.3389/fpsyg.2020.526288)
Supplement: Supplementary file 1 [file Table_1.DOCX]

**Supplementary Materials I: Stimuli and Details for Analyses 1a-d.**

**Selection of Stimuli**

**Study 1a. Selection of Stimuli**

The biggest hurdle in finding relatively standardized video material was the dearth of foreign affairs speeches given by women standing behind a podium in the halls of government. Thus, while most of the selected speeches were presented on the floor of the Senate or House (via C-SPAN), others were from presentations given at foundations and think tanks (e.g., the Heritage Foundation, Brookings Institute). Leaders were elected, office holders at the time (except for former Congresswoman Bachmann).

The selected political leaders for Study 1a were Senator Chris Murphy (D-CT), Representative Jim Himes (D-CT), Senator Kristen Gillibrand (D-NY), Senator Claire McCaskill (D-MI), Senator Marco Rubio (R-FL), Senator Rob Portman (R-OH), Former Representative Michele Bachmann (R-MN), and Senator Lisa Murkowski (R-AK).

**Study 1b. Selection of Stimuli**

Video stimuli were captured from statements made from the floor of the U.S. Senate to help standardize them (following ten Brinke et al., 2015). The original videos were extracted from the personal YouTube channels of 24 Senators randomly chosen from pools of male and female, Republican and Democratic leaders from 113^th^ and/or 114^th^ Senate. Randomness was restricted: politicians with regular, high profile media appearances were rejected to avoid participants’ pre-existing biases towards or against them. We omitted six senators from the pool due to their frequent presence in the news media. The six were: John McCain, Marco Rubio, Mitch McConnell, Harry Reid, Barbara Boxer, and Ted Cruz. By excluding well-known leaders, however, we may have inadvertently screened out some charismatic politicians.

**Study 1c and 1d. Selection of Stimuli (see text)**

**The Data Exclusion Rule: Studies 1a-b.**

In models with exclusions, ratings for a politician were excluded if the participant indicated that they recognized the politician (in the yes/no response option) *and* if they typed the name the politician in the subsequent, free-response option. The participant was categorized as correctly identifying the politician if the participant listed the politician’s first and/or last name correctly (as long as spelling was recognizable), or if the participant listed both the first and last name and it was very close to correct (e.g. “Michelle Bach” would be counted for Michelle Bachmann but “Buchanan” would not be counted as correct). If participants indicated “yes” to the recognition option but did not provide a name, they were categorized as not recognizing the politician.

**Primary analytic approach & justification: Studies 1a-b.**

Because each participant made multiple ratings of the key variables for multiple politicians, there was non-independence due to both participant and target (i.e., politician). Additionally, based on the exclusion criteria (above) as well as scattered, missing ratings, there were different sample sizes for each political leader who was rated. Linear mixed-effects models enabled us to account for the complex structures of non-independence in the data due to these factors and allowed us to assign greater weight to estimates made based on a larger number of observations. For these reasons, we used mixed effects-models to examine the relationships among receptivity, formidability, trustworthiness, and charisma.

**Overview of rationale for ancillary analyses of potential moderators: Studies 1a and 1b.**

Among the factors that might moderate the hypothesized relationships between receptivity, formidability, and charisma are leader gender, party (Democrat or Republican), and speech topic (domestic or foreign affairs). Specifically, stronger, positive relationships between receptivity and charisma might arise for female leaders because gender stereotypes highlight communal and caregiving capacities. The receptivity-charisma link may be stronger for Democratic than Republican leaders, reflecting the Democratic party’s association with communal, public good policies such as health care and education. For all political leaders, domestic affairs are more likely than foreign affairs to emphasize communal messages, and the nonverbal receptivity signals that go with them. The reverse might be true for the formidability-charisma relationship; male leaders, Republicans, and foreign affairs topics are likely to draw out displays of formidability, and strengthen the formidability-charisma link. These possibilities were explored by including their measurements in separate models.

**Additional Analyses and Summary Tables for Studies 1a-d.**

**Study 1a. Model 1 tests for moderators (exclusion rule in place):**

Separate models tested whether relationships between receptivity, formidability, and charisma were moderated by the party of the target (politician), the gender of the target, or the type of speech the participant watched when they rated the target. To test these possibilities, three, additional mixed-effects models were run that added the appropriate interactions needed to test these possibilities. None of the interactions were statistically significant. The results of these tests appear in Tables S2-4.

**Gender of Target**. Gender was coded as -.5 for female targets and +.5 for male targets. To test for moderation of effects by gender, I included target gender and its interactions with each other predictor variable in the model. There was no evidence of either overall effects of target gender on ratings of charisma or evidence that gender moderated the effects of receptivity, formidability, or trustworthiness on charisma (all ps > .19). See Table S-1.

**Party of Target (Democratic/Republican)**. The same approach was taken to see if the target’s party affiliation moderated any effects. Party was coded as -.5 = Republican and +.5 = Democrat. There was no overall effect of party, b = -0.26, t(7) = -0.89, p = 0.40 , and no evidence of moderating effects (all *p*s > .52). See Table S-2.

**Speech Topic (Domestic Policy/Foreign Affairs)**. The same approach was taken to examine whether speech topic moderated the effects. Domestic was coded as -.5 and Foreign as +.5. There was no evidence of an effect of topic on charisma ratings, b = 0.17, t(187.2) = .9, *p* = .37 and no evidence that topic moderated other effects, all *p*s > .34. See Table S-3.

**Study 1b. Tests for moderators (with exclusions)**

As for Study 1a, additional models probed the potential moderating effects of political leaders’ gender, party, and speech content. These analyses appear in Tables S-4 through S-6.

Results for the model examining gender and its interactions with each predictor in the model uncovered no evidence of either overall effects of politicians’ gender on ratings of charisma or evidence that gender moderated the effects of receptivity, formidability, or trustworthiness on charisma (all *p*s > .41) (see Table S-4).

A similar approach was taken to see whether political leaders’ party affiliation moderated any effects, though it was never expressly revealed to participants (see Table S-3). Party was coded -.5 for Republican and +.5 for Democrat. Results of this model indicated that Party moderated the relationship between Receptivity and Charisma, b = .06, *t*(4526.9) = 1.98, *p* = .04. Two additional models examined this interaction in greater detail, using dummy coding to elucidate the simple effect of Receptivity separately on Democratic and Republican political leaders. The relationship between Receptivity and Charisma was significant and positive for both Democrat and Republican politicians, but was stronger for Democratic leaders, b = .52, t(783.73) = 26.63, p <.002, than for Republican leaders, b = .45, t(1645)= 15.42, p < .001.

To examine whether speech content acted as a moderator, domestic speeches were coded as -.5 and foreign speeches were coded as +.5. Participants had no explicit awareness of speech content; differences attached to speech content can only have been derived through nonverbal information. There was evidence that speech topic moderated the receptivity-charisma relationship, b = -.09, t(520.9) = -2.58, *p* = 0.01. As before, the simple effect of Receptivity was evaluated separately for domestic and foreign speeches. The receptivity-charisma relationship for domestic speeches was a larger and more positive than for foreign affairs speeches, b = .55, t(610.1) = 22.55, *p* < .001 and b = .46, t(622.4) = 19.12, *p* < .001, respectively.

The overall analysis for Study 1b was rerun without the exclusion rule in place. Results did not change. Next, models probing moderators were rerun without the exclusion rule in place. The same basic pattern of findings held even when participants who correctly identified the target were included. See Tables S-7 through S-10 for these summary results.

**Summary of Moderator Effects for Study 1a and b.**

The overall results of Studies 1a and 1b supported Hypothesis 1: impressions of Receptivity and Formidability independently predicted Charisma. Ancillary tests of potential moderators of these relationships yielded two interesting findings. Though participants were uninformed about leader party and heard no speech content, these variables nevertheless moderated how charisma was perceived in Study 1b: The receptivity-charisma link was greater for Democratic than Republican leaders, and stronger for all leaders when they spoke about Domestic compared to Foreign issues. There was no evidence that political leaders’ Gender modified results.

| **Table S-1. Test of moderation of effect by Target Gender**  **Study 1a** | | |  |  |
| --- | --- | --- | --- | --- |
| Fixed Effects | b | df | t | p |
| Intercept | 0.04 | 8.9 | 0.21 | 0.84 |
| Receptivity | 0.39 | 13.4 | 7.88 | <.001 |
| Formidability | 0.53 | 676.7 | 15.12 | <.001 |
| Trustworthiness | 0.05 | 665.1 | 1.44 | 0.15 |
| Gender | -0.35 | 7.4 | -1.25 | 0.25 |
| Receptivity X Gender | 0.9 | 9.5 | 1.42 | 0.19 |
| Formidability X Gender | -0.06 | 2176 | -0.15 | 0.89 |
| Trustworthiness X Gender | 0 | 2107 | -0.01 | 0.99 |
| Random Effects | Variance |  |  |  |
| intercept: target | 0.33 |  |  |  |
| receptivity: target | 0.07 |  |  |  |
| intercept: participant | 0.54 |  |  |  |
| receptivity: participant | 0.24 |  |  |  |
| formidability: participant | 0.28 |  |  |  |
| trustworthiness: participant | 0.37 |  |  |  |
|  |  |  |  |  |

| **Table S-2. Test of moderation of effect by Target Political Party**  **Study 1a** | | |  |  |
| --- | --- | --- | --- | --- |
| Fixed Effects | b | df | t | p |
| Intercept | 0 | 8.4 | 0.02 | 0.99 |
| Receptivity | 0.41 | 11.9 | 7.7 | <.001 |
| Formidability | 0.42 | 714.5 | 14.5 | <.001 |
| Trustworthiness | 0.05 | 689.2 | 1.45 | 0.15 |
| Party | -0.26 | 7 | -0.89 | 0.4 |
| Receptivity X Party | 0.05 | 8.8 | 0.67 | 0.52 |
| Formidability X Party | 0.01 | 210 | 0.34 | 0.74 |
| Trustworthiness X Party | 0 | 204.3 | -0.1 | 0.92 |
| Random Effects | Variance |  |  |  |
| intercept: target | 0.36 |  |  |  |
| receptivity: target | 0.08 |  |  |  |
| intercept: participant | 0.55 |  |  |  |
| receptivity: participant | 0.24 |  |  |  |
| formidability: participant | 0.29 |  |  |  |
| trustworthiness: participant | 0.27 |  |  |  |

| **Table S-3. Test of moderation of effect by Topic of Speech**  **Study 1a** | | |  |  |
| --- | --- | --- | --- | --- |
| Fixed Effects | b | df | t | p |
| Intercept | -0.14 | 11 | -0.87 | 0.4 |
| Receptivity | 0.44 | 16.6 | 11.49 | <.001 |
| Formidability | 0.53 | 320.4 | 19.15 | <.001 |
| Trustworthiness | 0.05 | 319.8 | 1.77 | 0.08 |
| Topic | 0.17 | 187.2 | 0.9 | 0.37 |
| Receptivity X Topic | 0 | 314.9 | 0.06 | 0.95 |
| Formidability X Topic | -0.01 | 316.9 | -0.15 | 0.88 |
| Trustworthiness X Topic | -0.05 | 317 | -0.95 | 0.34 |
| Random Effects | Variance |  |  |  |
| intercept: target | 0.35 |  |  |  |
| receptivity: target | 0.08 |  |  |  |
| intercept: participant | 0.55 |  |  |  |
| receptivity: participant | 0.24 |  |  |  |
| formidability: participant | 0.29 |  |  |  |
| trustworthiness: participant | 0.27 |  |  |  |

**Table S-4. Test of moderation of effect by Target Gender**

**Study 1b: With exclusions**

| Fixed Effects | b | df | t | p |
| --- | --- | --- | --- | --- |
| Intercept | 0 | 64.6 | 0.01 | 0.99 |
| Receptivity | 0.5 | 867 | 24.77 | <.001 |
| Formidability | 0.44 | 52.3 | 18.67 | <.001 |
| Trustworthiness | 0.03 | 149.3 | 1.84 | 0.07 |
| Gender | 0.01 | 48.1 | 0.04 | 0.97 |
| Receptivity X Gender | 0 | 4506 | -0.01 | 0.99 |
| Formidability X Gender | -0.02 | 21.5 | -0.49 | 0.63 |
| Trustworthiness X Gender | 0.03 | 102.1 | 0.82 | 0.41 |
| Random Effects | Variance |  |  |  |
| intercept: target | 0.14 |  |  |  |
| formidability: target | 0.05 |  |  |  |
| trustworthiness: target | 0.02 |  |  |  |
| intercept: participant | 0.47 |  |  |  |
| receptivity: participant | 0.24 |  |  |  |
| formidability: participant | 0.25 |  |  |  |
| trustworthiness: participant | 0.19 |  |  |  |

| **Table S-5. Test of moderation of effect by Target Political Party**  **Study 1b:** W**ith exclusions** | | | |  |
| --- | --- | --- | --- | --- |
| Fixed Effects | b | df | t | p |
| Intercept | 0.03 | 66.7 | 0.36 | 0.72 |
| Receptivity | 0.48 | 850.6 | 24.19 | <.001 |
| Formidability | 0.45 | 54.4 | 19.65 | <.001 |
| Trustworthiness | 0.04 | 152.8 | 2.08 | 0.04 |
| Party | -0.09 | 48.5 | -0.71 | 0.48 |
| Receptivity X Party | 0.06 | 4526.9 | 1.98 | 0.04 |
| Formidability X Party | -0.07 | 31 | -1.76 | 0.09 |
| Trustworthiness X Party | 0.01 | 99.4 | 0.23 | 0.82 |
| Random Effects | Variance |  |  |  |
| intercept: target | 0.14 |  |  |  |
| formidability: target | 0.05 |  |  |  |
| trustworthiness: target | 0.02 |  |  |  |
| intercept: participant | 0.47 |  |  |  |
| receptivity: participant | 0.24 |  |  |  |
| formidability: participant | 0.25 |  |  |  |
| trustworthiness: participant | 0.19 |  |  |  |

| **Table S-6. Test of moderation of effect by Topic of Speech**  **Study 1b: With exclusions** | | |  |  |
| --- | --- | --- | --- | --- |
| Fixed Effects | b | df | t | p |
| Intercept | 0 | 74.9 | -0.03 | 0.98 |
| Receptivity | 0.5 | 627.4 | 28.01 | <.001 |
| Formidability | 0.43 | 58.3 | 21.2 | <.001 |
| Trustworthiness | 0.04 | 162.7 | 2.53 | 0.01 |
| Topic | 0.07 | 243.7 | 0.61 | 0.54 |
| Receptivity X Topic | -0.09 | 520.9 | -2.58 | 0.01 |
| Formidability X Topic | 0.06 | 488.1 | 1.76 | 0.08 |
| Trustworthiness X Topic | -0.01 | 507.7 | -0.3 | 0.77 |
| Random Effects | Variance |  |  |  |
| intercept: target | 0.14 |  |  |  |
| formidability: target | 0.05 |  |  |  |
| trustworthiness: target | 0.02 |  |  |  |
| intercept: participant | 0.48 |  |  |  |
| receptivity: participant | 0.23 |  |  |  |
| formidability: participant | 0.25 |  |  |  |
| trustworthiness: participant | 0.19 |  |  |  |

**Analyses for Study 1b repeated without the exclusion rule in place.**

| **Table S-7. Test of Model 1**  **Study 1b: No exclusions** | |  |  |  |
| --- | --- | --- | --- | --- |
| Fixed Effects | b | df | t | p |
| Intercept | 0.27 | 498.7 | 1.2 | 0.23 |
| Receptivity | 0.53 | 1777 | 5.9 | <.001 |
| Formidability | 0.29 | 928.7 | 4.57 | <.001 |
| Trustworthiness | -0.02 | 1340 | -0.35 | 0.73 |
| Receptivity X Formidability | 0.02 | 1913 | 0.84 | 0.4 |
| Formidability X Trust | 0.03 | 1483 | 2.1 | 0.04 |
| Receptivity X Trust | -0.01 | 2181 | -0.51 | 0.61 |
| Recep. X Formid. X Trust | 0 | 1642 | -0.71 | 0.48 |
| Random Effects | Variance |  |  |  |
| intercept: target | 0.15 |  |  |  |
| formidability: target | 0.05 |  |  |  |
| trustworthiness: target | 0.02 |  |  |  |
| intercept: participant | 0.47 |  |  |  |
| receptivity: participant | 0.23 |  |  |  |
| formidability: participant | 0.25 |  |  |  |
| trustworthiness: participant | 0.19 |  |  |  |

**Tests of Potential Moderators**

**Study 1b: No Exclusions**

Models probing moderators were rerun, this time without the exclusion rule in place. The same basic pattern of findings held even when participants who correctly identified the target were included. See Tables S-8 through S-10.

| **Table S-8. Test of moderation of effect by Target Gender,**  **Study 1b: No exclusions** | | | |  |
| --- | --- | --- | --- | --- |
| Fixed Effects | b | df | t | p |
| Intercept | 0 | 60.6 | -0.01 | 0.99 |
| Receptivity | 0.5 | 886.3 | 25.35 | <.001 |
| Formidability | 0.44 | 54.4 | 19.31 | <.001 |
| Trustworthiness | 0.03 | 163.5 | 1.76 | 0.08 |
| Gender | 0.03 | 43.6 | 0.2 | 0.84 |
| Receptivity X Gender | 0 | 4694 | 0.08 | 0.94 |
| Formidability X Gender | -0.02 | 31.4 | -0.41 | 0.69 |
| Trustworthiness X Gender | 0.01 | 109 | 0.47 | 0.64 |
| Random Effects | Variance |  |  |  |
| intercept: target | 0.15 |  |  |  |
| formidability: target | 0.05 |  |  |  |
| trustworthiness: target | 0.02 |  |  |  |
| intercept: participant | 0.49 |  |  |  |
| receptivity: participant | 0.23 |  |  |  |
| formidability: participant | 0.25 |  |  |  |
| trustworthiness: participant | 0.19 |  |  |  |

| **Table S-9. Test of moderation of effect by Target Political Party,**  **Study 1b No exclusions** | | | | |
| --- | --- | --- | --- | --- |
| Fixed Effects | b | df | t | p |
| Intercept | 0.03 | 64.3 | 0.36 | 0.72 |
| Receptivity | 0.48 | 882.8 | 24.69 | <.001 |
| Formidability | 0.46 | 57.7 | 20.29 | <.001 |
| Trustworthiness | 0.03 | 174.3 | 1.9 | 0.06 |
| Party | -0.07 | 45.4 | -0.56 | 0.58 |
| Receptivity X Party | 0.06 | 4776 | 1.88 | 0.06 |
| Formidability X Party | -0.07 | 31.3 | -1.74 | 0.09 |
| Trustworthiness X Party | 0 | 112.4 | 0.08 | 0.94 |
| Random Effects | Variance |  |  |  |
| intercept: target | 0.15 |  |  |  |
| formidability: target | 0.05 |  |  |  |
| trustworthiness: target | 0.02 |  |  |  |
| intercept: participant | 0.49 |  |  |  |
| receptivity: participant | 0.24 |  |  |  |
| formidability: participant | 0.25 |  |  |  |
| trustworthiness: participant | 0.19 |  |  |  |

| **Table S-10. Test of moderation of effect by Topic of Speech,**  **Study 1b: No exclusions** | | | |  |
| --- | --- | --- | --- | --- |
| Fixed Effects | b | df | t | p |
| Intercept | 0 | 71.9 | 0.05 | 0.96 |
| Receptivity | 0.5 | 644.7 | 28.61 | <.001 |
| Formidability | 0.44 | 61.7 | 21.89 | <.001 |
| Trustworthiness | 0.04 | 182 | 2.25 | 0.03 |
| Topic | 0.06 | 248.7 | 0.54 | 0.59 |
| Receptivity X Topic | -0.08 | 530.4 | -2.4 | 0.02 |
| Formidability X Topic | 0.06 | 491.8 | 1.64 | 0.1 |
| Trustworthiness X Topic | -0.01 | 502.9 | -0.29 | 0.77 |
| Random Effects | Variance |  |  |  |
| intercept: target | 0.14 |  |  |  |
| formidability: target | 0.05 |  |  |  |
| trustworthiness: target | 0.02 |  |  |  |
| intercept: participant | 0.5 |  |  |  |
| receptivity: participant | 0.23 |  |  |  |
| formidability: participant | 0.25 |  |  |  |
| trustworthiness: participant | 0.19 |  |  |  |
