# Supplementary material for: Charismatic Nonverbal Displays by Leaders Signal Receptivity and Formidability, and Tap Approach and Avoidance Motivational Systems
Source: Front Psychol. 2020 Oct 22;11:526288. doi: 10.3389/fpsyg.2020.526288 (PMC7643026; doi:10.3389/fpsyg.2020.526288)
Supplement: Supplementary file 2 [file Table_2.DOCX]

Supplementary Materials II. Details of Coding Procedure

OPERATIONAL DEFINITIONS OF CODED VARIABLES:

**VARIABLE HOW CODED (OPERATIONALIZED)**

Smiling An upward turn of the lip corners (Stewart & Dowe 2013); number of times, duration

Hand faced towards body: To move hand(s) with the condition that the audience

cannot see the palm

Hand faced away from body: To move hand(s) with the condition that the audience can

see the palm

One Hand: To move one hand from the baseline resting position while

the other hand remains at rest

Two Hands: To move two hands from the baseline resting position

Brow Frown:

To lower eyebrows’ downwards closer to the eyes

and the brow’s midline.

Brow Raise:

To raise the eyebrows upwards in a prompt manner

Eye blink: NUMBER OF TIMES the closing of both eyelids

General Affective Facial Display Facial display of negative or positive emotional tone rated

on a 5

point scale

1 = extremely negative

2 = negative

3 = neutral

4 = positive

5 = extremely positive

----------------------------------------------------------------------------------------------------------------------------

Number of hand gestures arm/hand movement between pauses (gesture is counted

as a single gesture until it stops/pauses)

Intensity of gestures individual intensity rating (1-4) for each gesture; overall

intensity rating is the average (rate of onset, strong/weak)

1 (not intense) - 4 (very intense)

**(lips pursed, pout & shake head = numbers recorded are the number of times it occurred over the course of the video)

Lips Pursed - to press your lips together and outward ex. because you are angry and/or are think


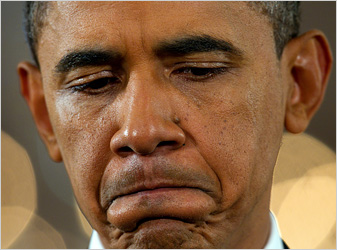


**Pout** - to push ones lips, especially bottom lip, forward as an expression of annoyance, etc.


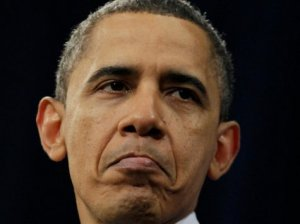


**Shake Head**- to move ones head from left to right,, usually in disapproval


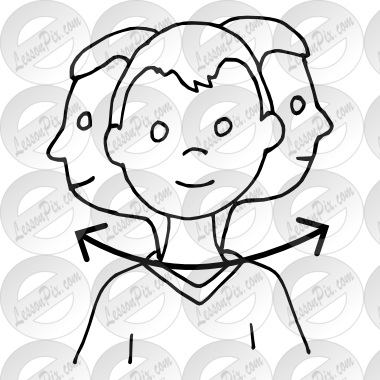


**Variable -** Gaze

**Operational Definition -** time spent looking up at the audience

Hands

Pointing= extending one finger while the other four fingers are curled into the palm, each “jab” of the finger is considered one point

Fisting= extending the hand while all five fingers are curled into the palm, each “jab” or pound of the first is considered one fist


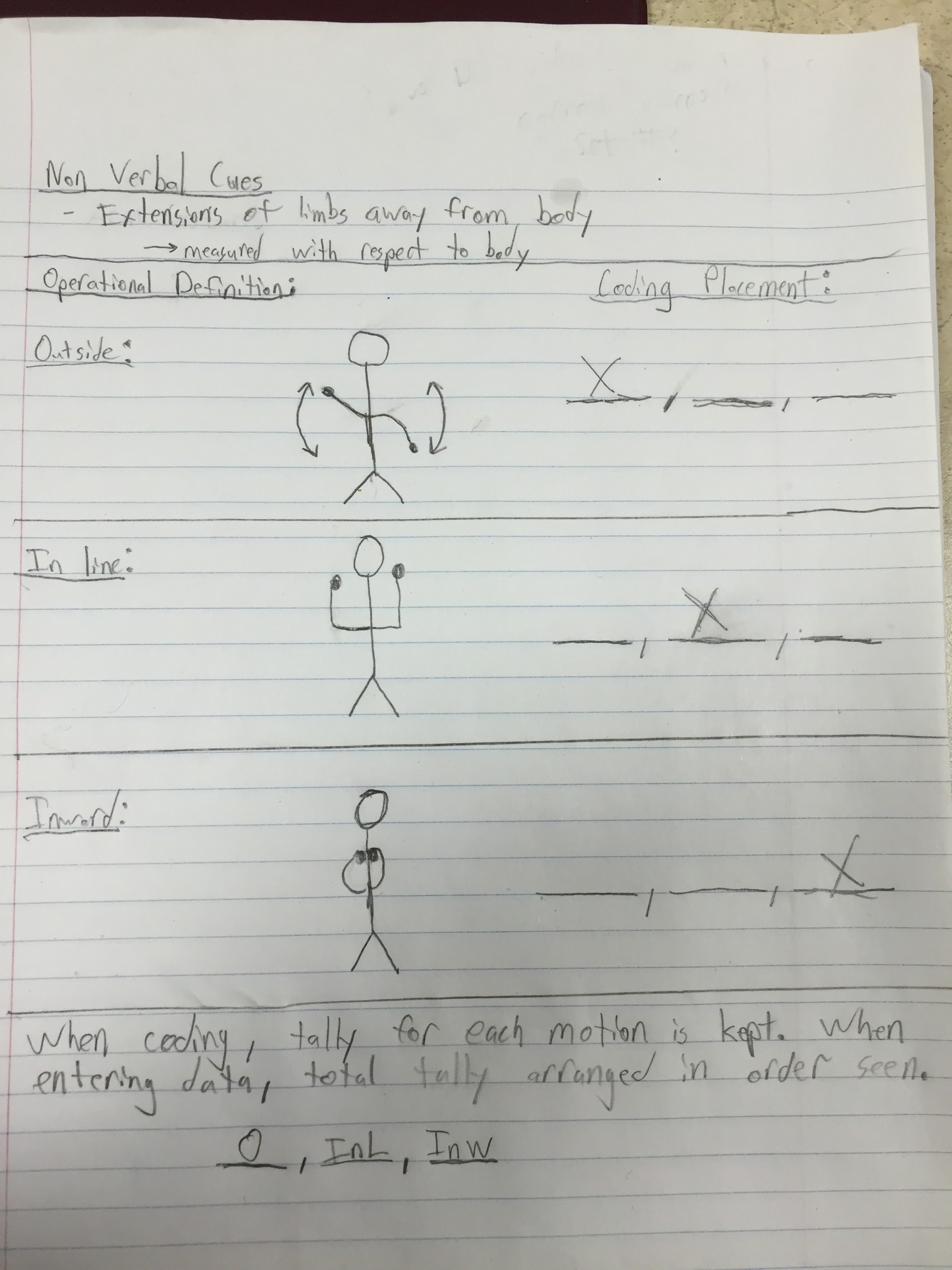


Any sort of motion includes at least both hand and forearm, not just hand movement.

For outside the body, hands and forearms outside the hips; general direction of movement outwards.

For in line with body, arm motions pointed towards audience. As long as upper arm/elbow are parallel with up-and-down body axis.

For inwards, hands and forearms brought in towards the center of the body.

Orchestrating: Repetitive, flowing, gesture that continues over seconds.
